# Tunable Switching Mechanisms in HfZrO_2_‐Based Tunnel Junctions for High‐Performance Synaptic Arrays

**DOI:** 10.1002/advs.202516478

**Published:** 2026-02-23

**Authors:** Jiwon You, Jeong‐Han Kim, Minsuk Song, Been Kwak, Eun Chan Park, Manh‐Cuong Nguyen, Wonjun Shin, Jangsaeng Kim, Daewoong Kwon

**Affiliations:** ^1^ Department of AI Semiconductor Engineering Hanyang University Seoul Republic of Korea; ^2^ Department of Electrical Engineering Hanyang University Seoul Republic of Korea; ^3^ Department of Nanoscale Semiconductor Engineering Hanyang University Seoul Republic of Korea; ^4^ 3‐D Convergence Center Inha University Incheon Republic of Korea; ^5^ Department of Semiconductor Convergence Engineering Sungkyunkwan University Suwon Republic of Korea; ^6^ Department of Electronic Engineering Sogang University Seoul Republic of Korea; ^7^ Department of System Semiconductor Engineering Sogang University Seoul Republic of Korea

**Keywords:** ferroelectric tunnel junctions, HfZrO_2_, hybrid switching, large‐scale crossbar array, oxygen vacancies, vision transformer

## Abstract

Strategic optimization of ferroelectric tunnel junctions (FTJs) is critical for advancing nonvolatile memory and neuromorphic computing technologies. In this work, we present a comprehensive study on materials and structural engineering to enable scalable hybrid‐switching FTJ arrays. We systematically manipulated oxygen vacancy (V_O_) concentrations in HfZrO_2_ (HZO) films through strategic choices of bottom electrodes and interfacial layers, achieving three distinct operational modes: pure ferroelectric switching, defect‐modulated switching, and combined hybrid switching. Our optimized devices demonstrate exceptional tunneling electroresistance (TER) performance: Mo bottom electrodes achieve a TER ratio of around 10^2^, while Mo/Ti bottom electrodes attain TER to over 10^4^. Lower‐leakage ferroelectric switching and enhanced polarization stability are observed with Mo bottom and ZrO_2_ interlayers, while V_O_‐driven resistive contributions from Ti electrodes amplify TER in hybrid devices. Utilizing these optimized parameters, we fabricated a 42 × 42 FTJ array demonstrating uniform multi‐level conductance modulation. The fabricated FTJ array was integrated into an in‐memory Vision Transformer (ViT) architecture, successfully performing stable and energy‐efficient parallel vector–matrix multiplication (VMM) operations despite device variability. This work shows that precisely engineered, large‐area hybrid‐switching FTJ arrays can provide a scalable and energy‐efficient hardware platform for next‐generation memory and neuromorphic systems.

## Graphical abstract

This work demonstrates hybrid switching in engineered HZO‐based FTJs, enabled by controlled interlayer design and oxygen scavenging dynamics. The combined switching mechanism produces robust multilevel conductance states in large crossbar arrays, offering a materials‐driven pathway toward scalable in‐memory computing with enhanced tunability and stability.

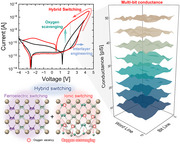

## 1 Introduction

Extensive research efforts have been dedicated to harnessing ferroelectricity for memory applications, leveraging its rapid switching speed and low program/erase bias voltages, in contrast to conventional charge‐trap‐based flash memories. These efforts have focused on refining the material properties of thin films, particularly perovskites with octahedral oxygen structures such as BaTiO_3_ [1, 2, 3]. The exploration has gained significant momentum with the discovery of ferroelectricity in hafnium oxide (HfO_2_)‐based thin films with fluorite‐structured binary oxides (fluorites) [4, 5, 6, 7, 8, 9]. This breakthrough fueled further research owing to its compatibility with complementary metal‐oxide semiconductor (CMOS) technology [10]. As a result, ferroelectric‐based memory has become a focal point of investigation for global memory companies, including industry giants such as Intel, Taiwan Semiconductor Manufacturing Company (TSMC), and Samsung Electronics [11, 12, 13]. In contemporary contexts, the scope of application of ferroelectric materials extends beyond traditional memory to neuromorphic computing [14, 15]. The unique characteristics of ferroelectricity, including its multi‐conductance and abrupt switching derived from domain‐based switching [16], enable its use in realizing synaptic and neuronal functionalities [17, 18, 19].

The most widely adopted structure for incorporating ferroelectric materials into memory or neuromorphic computing is the ferroelectric tunnel junction (FTJ) [20, 21, 22, 23, 24, 25]. This structure has a straightforward design consisting of a ferroelectric layer sandwiched between two electrodes. FTJs have garnered significant attention owing to their ease of fabrication and high degree of flexibility in adjusting and optimizing the material characteristics of ferroelectric films. Notably, the FTJ structure has also been integrated into ferroelectric field‐effect transistors and ferroelectric thin‐film transistors as a gate stack, making research on FTJs essential for the broader field of ferroelectric memory [26].

The operational principle of the FTJ is straightforward. The tunneling electroresistance (TER) is altered by polarization switching, which is influenced by the asymmetric energy barrier landscape between the top and bottom electrodes [27]. The ferroelectricity of polycrystalline fluorites is introduced through a non‐centrosymmetric orthorhombic phase (o‐phase) with space group Pca2_1_ [28, 29, 30]. Various methods have been employed to enhance the ferroelectricity of these films, including controlling the film thickness, doping with elements such as Si, Al, and Y [31, 32, 33], and inducing strain effects by modulating the material of metal electrodes with different thermal coefficients. However, reliability issues pose significant obstacles to commercialization, including retention instability caused by the depolarization field and weak endurance characteristics due to excessive field application at the ferroelectric/dielectric interface during program/erase cycling [34]. Accordingly, research efforts have been made to address these challenges by controlling oxygen vacancies through the different scavenging effects of metal electrodes and by optimizing the dielectric material and structure [34, 35, 36].

In these previous studies, it was assumed that the TER ratio in FTJs could be enhanced by increasing the remnant polarization [37]. According to this premise, the conduction within FTJs is regulated by the modulation of the tunneling barrier height, which is controlled by ferroelectric polarization. Nevertheless, recent research findings indicate that the carrier transport within HfO_2_ with a fluorite structure is affected by additional factors. These include the redistribution of oxygen vacancies and the formation/rupture of conductive filaments, a phenomenon well established by numerous studies delving into the working principles of resistive random‐access memory (RRAM) [38, 39, 40, 41, 42]. This prompted us to investigate whether FTJs function solely as devices for ferroelectric polarization switching. Recent reports have addressed this issue by presenting evidence of the coexistence of polarization switching and other resistive switching mechanisms within FTJs [39, 42, 43, 44, 45]. Consequently, future studies on FTJs should not only focus on augmenting the polarization of the film but also consider the coexistence of various resistive switching mechanisms within FTJs. However, research in this area is scarce, and the development of methods to control and optimize different switching mechanisms is of paramount importance.

Building on the preceding discussion, this study investigates the material and structural influences of FTJs on various resistive switching mechanisms. Herein, we present a method to control these mechanisms, primarily by manipulating the oxygen vacancy (V_O_) content within the ferroelectric film. The ferroelectric layer in this study consisted of HfZrO_2_ (HZO) situated between the top and dielectric/bottom electrodes. Diverse combinations of bottom electrodes and dielectric layers are employed, leading to the fabrication of devices that demonstrate either polarization switching only (ferroelectric‐based switching), defect‐modulated switching only (non‐ferroelectric switching), or a combination of both ferroelectric and non‐ferroelectric switching mechanisms. These findings underscore the substantial role played by oxygen vacancy redistribution within the film in determining the working principles of FTJs.

The manuscript is organized as follows. First, we explore the impact of the bottom electrode by examining FTJs fabricated on p^+^ silicon and a metal electrode (Mo). Our investigation revealed that the reduced oxygen scavenging effects of the Mo bottom electrode on the HZO layer mitigated the influence of oxygen vacancy redistribution, thereby creating a pure polarization switching device. We demonstrated that these FTJs offer the advantages of low off‐current. This stems from the suppression of the trap‐assisted tunneling via oxygen vacancies, making them highly beneficial for both memory and neuromorphic computations. Additionally, we revealed that the TER ratio of pure polarization‐switching FTJs can be further increased by inserting an additional ZrO_2_ layer as a dielectric, inducing more o‐phases in HZO [46]. This method compensates for the smaller TER ratio of pure polarization‐switching FTJs compared to FTJs with a mixed resistive switching mechanism. Finally, we presented design guidelines for controlling the oxygen‐scavenging effects of the bottom electrode by fabricating FTJs with different types of metal electrodes. An increased oxygen vacancy concentration within the HZO layer enhances polarization switching, contributing to a larger TER ratio. Moreover, this approach enables FTJs with metal bottom electrodes to exhibit an oxygen‐vacancy redistribution‐induced resistive switching mechanism when pronounced oxygen scavenging effects are present. Consequently, the combination of polarization enhancement and resistive switching, achieved by controlling the oxygen vacancy concentration in HZO, maximizes the TER ratio while minimizing off‐current.

By optimizing the materials and structures, while considering the diverse inherent switching mechanisms of FTJs, we propose a hybrid‐switching FTJ—combining polarization switching and resistive switching—as an artificial synaptic device. Furthermore, we demonstrate the feasibility of a large‐scale synaptic array composed of hybrid‐switching FTJs for neuromorphic computing applications, such as an in‐memory vision transformer (ViT) system, specifically optimized for large‐scale vision models [18, 47, 48].

## 2 Results and Discussion

### 2.1 Electrical Properties of Ferroelectric Tunnel Junctions

Previous studies have reported that the TER ratio of FTJs is governed by the ferroelectric switching of HZO under the applied voltage [49]. However, considering that the resistance of FTJs with a ferroelectric layer (FE) and an interlayer (IL) between the two electrodes is determined by the tunneling length depending on the stored polarization states in the FE, the continuous increase in TER is theoretically unexplainable because the tunneling length can no longer be modulated after the FE becomes completely polarized. Therefore, the TER ratio should be saturated at voltages beyond the coercive voltage (*V*_C_).

**FIGURE 1:**
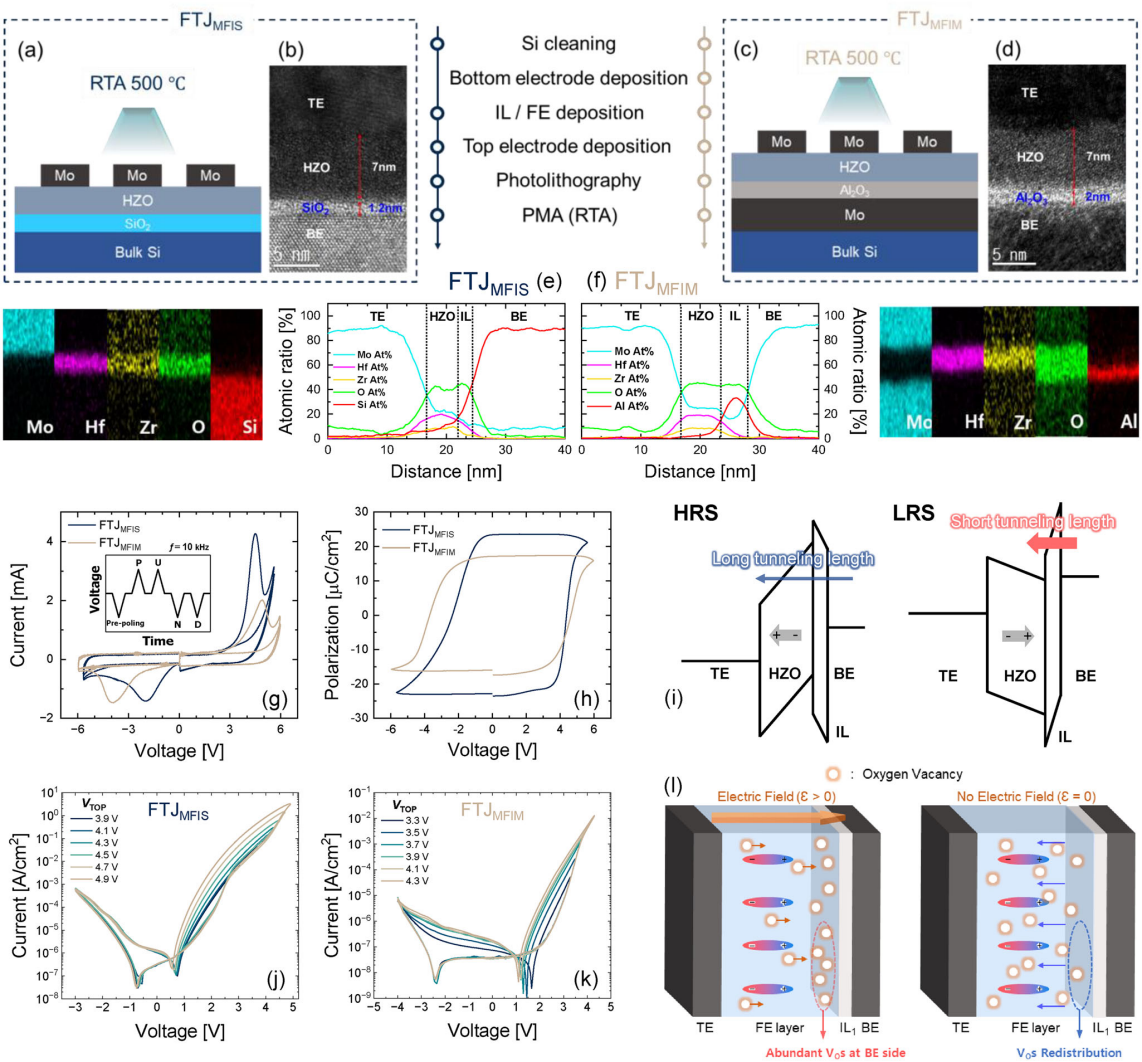
(a,c) Schematic illustrations of FTJs with different stack configurations: FTJ_MFIS_ and FTJ_MFIM_. Both structures use Mo top and bottom electrodes and 7 nm HZO ferroelectric layers, while FTJ_MFIM_ incorporates a 2 nm Al_2_O_3_ interlayer instead of 1.2 nm SiO_2_ in FTJ_MFIS_. RTA was performed at 500 °C to induce crystallization of HZO. (b,d) Cross‐sectional TEM images confirming the uniform deposition and thickness of each layer. (e,f) EDS elemental maps and line profiles validating the spatial distributions of constituent elements (Mo, Hf, Zr, O, Si in FTJs, and Al in FTJ_MFIM_). (g,h) *I–V* curves and *P–V* hysteresis curves of FTJ_MFIS_ and FTJ_MFIM_ were extracted through PUND measurements at a frequency of 100 kHz triangular pulses at room temperature (RT), indicating distinct ferroelectric switching with remanent polarizations (2*P*_r_) of ∼46 µC/cm^2^ and ∼34 µC/cm^2^. (i) Energy band diagrams illustrating switching mechanism from HRS to LRS governed by the redistribution of V_O_ and field‐induced polarization switching across the HZO. (j,k) *I–V* curves of FTJ_MFIS_ and FTJ_MFIM_ showing counterclockwise switching behavior under, indicating gradual polarization and memory window formation. The measurements were performed at RT with Δ*V* = 50 mV and *I*_comp_ (compliance current) = 1 mA. (l) Schematic of V_O_ redistribution mechanism, highlighting the role of built‐in electric field in controlling vacancy migration direction and magnitude.

To investigate the physical origin of these discrepancies in the TER ratio, two different types of FTJs were fabricated. As depicted in Figure 1a,c, one is an FTJ with metal‐FE‐IL‐Si stacks (FTJ_MFIS_), and the other is an FTJ with metal‐FE‐IL‐metal stacks (FTJ_MFIM_). The cross‐sectional transmission electron microscope (TEM) images show that FTJ_MFIS_ has Mo/ Hf_0.6_Zr_0.3_O_2_/SiO_2_/Si stacks (Mo/Hf_0.6_Zr_0.3_O_2_/Al_2_O_3_/Si stacks in Figure S1a), and FTJ_MFIM_ has Mo/HfZrO_2_(Hf: Zr = 2:1)/Al_2_O_3_/Mo stacks (Figure 1b,d). In addition, the energy dispersive spectroscopy (EDS) analysis in Figure 1e,f confirms the spatial distribution of each element throughout both FTJs.

First, the polarization switching currents were measured to confirm the ferroelectricity of the FTJs using positive‐up‐negative‐down (PUND) measurements after 1000 cycles of repeated triangular pulses to wake up the FE (Figure 1g). PUND measurements were performed by applying a 100 kHz triangular pulse to separate the ferroelectric switching current (*I*_SW_) from the non‐ferroelectric switching current, such as displacement and leakage currents. The extracted *I*_SW_–applied voltage (*I*_SW_–*V*) curve and the corresponding polarization‐voltage (*P–V*) curves are presented in Figure 1g,h, where remanent polarizations (*P*_r_) of 23 and 17 µC/cm^2^ were confirmed for FTJ_MFIS_ and FTJ_MFIM_, respectively.

Subsequently, hysteretic tunneling currents (*I*_T_) were measured to verify the memory properties of the FTJs. For the DC *I–V* measurement, a bias was applied to the top electrode and double‐swept in the forward (from − *V*_Max_ to + *V*_Max_) and reverse (from + *V*_Max_ to − *V*_Max_) directions; here, *V*_Max_ denotes the maximum top electrode voltage reached in each double sweep. In FTJs with an IL, the electric field applied to the IL changes depending on the polarization states of the FE, thereby modulating the tunneling length for Fowler–Nordheim (F‐N) tunneling [50, 51, 52, 53]. Tunneling length or tunneling current modulation caused by the change in polarization status is converted directly to a change in resistance, which is defined as TER. Thus, as shown in Figure 1i, at the beginning of forward voltage sweeping, the FE is polarized by − *V*_Max_ (long tunneling length), and the FTJ is in a high‐resistance state (HRS). In contrast, the starting + *V*_Max_ in reverse sweeping drives down the polarization (short tunneling length), and the FTJ has a low‐resistance state (LRS) [27]. Figure 1j,k shows the hysteretic *I*_T_ curves of the FTJ_MFIS_ and FTJ_MFIM_, respectively. Counterclockwise *I*_T_ hysteresis was observed for both FTJs, which indicates the TER ratio induced by polarization switching.

However, there were two significant differences between the two: (1) The TER ratio of FTJ_MFIS_ continues to increase with increasing *V*_Max_, whereas that of FTJ_MFIM_ tends to saturate. (2) While FTJ_MFIS_ has a clear *I*_T_ difference between the LRS and HRS when the *I*_T_s are extracted at read voltages (*V*_READ_) larger than 4 V, FTJ_MFIM_ has a negligible *I*_T_ difference. In Figure S2a,b, these *I*_T_ and TER ratio differences can be observed when the *I*_T_s extracted from the measured hysteretic *I*_T_ curves were converted to TER values, and the TER ratios [(I_LRS_‐I_HRS_)/I_HRS_] were calculated with respect to *V*_READ_ for various *V*_Max_ sweeping ranges. If the TER ratio is determined only by polarization switching, the TER ratio of the FTJs should not continue to increase after the FE is fully switched. However, the TER ratio of FTJ_MFIS_ increased without saturation. In addition, it increased abruptly from *V*_Max_ = ∼4 V, unlike FTJ_MFIM_ where the TER ratio tended to saturate with increasing *V*_Max_ as indicated. This difference can be explained by the *I*_T_ of forward sweeping abruptly increasing again around *V*_Max_ = 4 V, which increases the *I*_T_ of backward sweeping, indicating that the resistance of FTJ_MFIS_ is affected by the polarization state and an additional factor. To confirm that the observed resistive switching in FTJ_MFIS_ is not specific to the SiO_2_ interlayer, we fabricated an additional FTJ_MFIS_ device using Al_2_O_3_ as the interlayer. As shown in Figure S1b–d, the FTJ_MFIS_ also exhibited a continuously increasing memory window and a sharp rise in TER ratio beyond *V*_Max_ = 4.5 V, indicating that resistive switching occurs even with an Al_2_O_3_ interlayer. These results support that the resistive component in FTJ_MFIS_ arises from the MFIS structure itself, particularly the use of a silicon bottom electrode, rather than from the specific interlayer material.

To investigate the origin of the abnormal increase in the TER ratio of FTJ_MFIS_, resistive memory (ReMem) was fabricated using the same fabrication process as FTJ_MFIS_ except for post‐metal annealing (PMA) at 400°C, which is not high enough to form ferroelectricity (Figure S3a). The X‐ray diffraction (XRD) analysis (Figure S3d–f) and PUND measurements (Figure S3b) confirmed that the HZO film was still in an amorphous state after the low‐temperature PMA, and ferroelectricity did not exist in ReMem, whereas the HZO films in FTJ_MFIS_ and FTJ_MFIM_ underwent crystallization, resulting in the emergence of ferroelectricity. Because ReMem cannot undergo polarization switching, only an additional factor is expected to modulate its resistance. Figure S3c shows that the counterclockwise TER appears owing to the hysteretic DC *V*_Max_ sweeping, although ReMem does not exhibit ferroelectricity. Therefore, it is assumed that this TER ratio originates from the resistive switching of HZO, which occurs in HfO_2_‐based RRAM [39, 42]. More specifically, it results from a change in the potential barrier height due to the bias‐induced accumulation of oxygen vacancies (V_O_) at the interface between the IL and HfO_2_‐based dielectric [56]. Therefore, the TER ratios of FTJ_MFIM_ and ReMem are determined only by polarization switching or resistive switching of HZO, respectively, whereas that of FTJ_MFIS_ is affected by the coexistence of polarization and resistive switching in HZO. Recently, it has been reported that the TER ratio of FTJs with pure HfO_x_ FE is also determined by the sequential resistance changes induced by polarization switching (low‐bias region) and resistive switching (high‐bias region) of HfO_x_ [42]. Importantly, FTJ_MFIS_ with HZO FE exhibited a similar tendency. As shown in Figure S4a, *I*_T_ starts to flow by trap‐assisted tunneling (TAT) and F‐N tunneling. We define two thresholds for clarity: *V*_th,1_—the onset of the first current increase due to polarization‐switching (≈ 2 V), and *V*_th,2_—the onset of the second current increase due to resistive switching (≈ 4 V). Consistently, the first current increase appears at *V*_th,1_, followed by a steeper rise beyond *V*_th,2_. To verify the contribution of polarization switching, we additionally performed a unidirectional DC double sweep (0 to 5 V) to suppress negative‐bias‐induced polarization reversal. Under this condition, the distinct current increase around *V*_READ_ ≈ 2–3 V is largely diminished with an elevated HRS, while the LRS remains nearly unchanged, resulting in a reduced TER in this range. In addition to V_O_‐redistribution effects, electrode‐induced factors may also play a role [57, 58, 59]. These include the work function difference between Mo and p^+^‐Si and the stabilization of the ferroelectric o‐phase, which can further modulate the initial barrier tilt and internal field, contributing to differences in TER and *P*_r_ between FTJ_MFIS_ and FTJ_MFIM_.

To clarify the physical origin of the difference between FTJ_MFIM_ and FTJ_MFIS_, the spatial distribution and concentration of V_O_s inside HZO were extracted because it is well‐known that V_O_s are strongly related to ferroelectricity formation in FTJs [60, 61] and resistive switching in RRAMs [45, 56, 66]. To extract the V_O_s ratio, X‐ray photoelectron spectroscopy (XPS) depth profile analysis was performed (Figure S5a–c), which makes it possible to analyze the oxygen vacancies in the bulk of HZO. Up to 60 s etching time is considered the region of the HZO film because the atomic concentrations of Hf4f and Zr3d elements start to decrease, and those of Si2p and Al2p elements that comprise the interlayer start to increase from 60 s in all the devices. Therefore, the spectra were first corrected with respect to the C1s peak located at 284.8 eV. The oxygen vacancy ratio was then extracted from the XPS spectra of O1s in the HZO region for etching times ranging from 5 to 60 s. Figure S5d shows that FTJ_MFIM_ contains a smaller oxygen vacancy than FTJ_MFIS_ and ReMem.

From the analysis of the V_O_ ratio, the difference in the hysteretic *I*_T_ characteristics between FTJ_MFIS_ and FTJ_MFIM_ can be explained as follows: (1) V_O_s serve as trap sites to accelerate TAT, such that a large TAT current flows in the V_O_‐rich FTJ_MFIS_ before the first abrupt *I*_T_ increase by polarization switching (Figure S4a,c), leading to a reduction in the TER ratio. In contrast, in FTJ_MFIM_, a negligible TAT current is maintained owing to the reduced V_O_s, and the *I*_T_ abruptly increases by polarization switching (Figure S4b,d); thus, a larger TER ratio is achievable in the TAT region compared to FTJ_MFIS_ without resistive switching. (2) Only for FTJ_MFIS_, the second abrupt *I*_T_ increase (i.e., resistive switching) occurs owing to the redistribution of V_O_s (Figure S4e), whereas the resistive switching of HZO is not observed in FTJ_MFIM_ owing to the lack of V_O_s. More specifically, FTJ_MFIS_s are characterized by the gradual migration of V_O_ toward the FE/IL interface under the influence of an electric field. When this field is removed, these vacancies readily redistribute within the ferroelectric layer, as illustrated in Figure 1l. In contrast, FTJ_MFIM_s operate primarily through mechanisms of polarization reversal switching and depolarization. Furthermore, the different polarization behaviors shown in Figure 1h (FTJ_MFIS_ has a larger *P*_r_ and smaller coercive electric field than FTJ_MFIM_) can also be explained by the different numbers of oxygen vacancies in the FTJs. In hafnia‐based ferroelectric materials, oxygen vacancies are known to play an important role in influencing polarization behavior [60, 61]. However, prior studies have shown that other factors—such as o‐phase stabilization, grain size, dopant, annealing conditions, internal strain, deposition temperature, and thickness—can also contribute to the emergence and enhancement of ferroelectricity [31, 32, 33, 62, 63, 64, 65]. Therefore, we suggest that the improved *P*_r_ observed in FTJ_MFIS_s, which tend to be V_O_‐rich, is likely the result of combined effects of oxygen vacancies together with these additional structural and interfacial factors, rather than being solely attributable to oxygen vacancies.

### 2.2 Temperature Dependency and Conducting Mechanisms of FTJs

Hysteretic *I*_T_–*V*_Max_ curves were measured across various temperatures to investigate the conduction mechanisms of the FTJs. Figure S6a,c show that the TER ratios of FTJ_MFIS_ increase significantly with rising temperatures, despite a reduction in *P*_r_ (Figure S6b,e). In contrast, the TER ratios of FTJ_MFIM_ exhibit only slight changes, corresponding to an increase in *P*_r_ (Figure S6d,g). This opposite temperature dependence of Pr originates from different polarization screening conditions determined by the bottom electrode: in FTJ_MFIS_, less effective screening enhances depolarization‐driven polarization relaxation/back‐switching, whereas in FTJ_MFIM_, metal‐assisted screening suppresses depolarization and enables more complete switching, increasing *P*_r_. Notably, in FTJ_MFIS_, *I*_T_ remains relatively insensitive to temperature variations up to *V*_Max_ = ∼4 V. Beyond this threshold, the slope of *I*_T_ begins to change with temperature, becoming steeper at higher temperatures and resulting in temperature‐dependent fluctuations in the TER ratio. This behavior indicates a transition in the conduction mechanism from polarization‐driven tunneling to resistive switching. The tunneling current induced by polarization switching is relatively unaffected by temperature variations, whereas barrier height modulation due to oxygen vacancy redistribution (resistive switching) is highly sensitive to temperature changes.

ln(*I*/*E* ^2^) versus 1/*E* curves (F–N plot) plots were calculated for the LRS to separate the current flow mechanisms for the FTJs. According to the F‐N tunneling current Equation 1, the slope of the extracted curves should be constant regardless of the temperature if F‐N tunneling dominates the conduction mechanism [53].

$$\begin{array}{ccc} & & I_{FN,T}=\frac{q^{3}AV^{2}}{8\pi t^{2}hq\phi_{B}}\;exp\left[\frac{-8\pi t{\left(2qm_{T}^{*}\right)}^{1/2}}{3hV}\phi_{B}^{3/2}\right],\;\\ & & Slope_{FN,T}=\frac{-8\pi \sqrt{2qm_{T}^{*}\phi_{B}^{3}}}{3h} \end{array}$$

where *q* is the electronic charge, *A* is the dimension of devices, *t* is the dielectric thickness, *h* is Planck's constant, *Φ_B_* is the Schottky barrier height, and *m* ^*^_T_ is the tunneling effective mass in a dielectric (*m* ^*^_T_ ≈ 2m_0_) [54]. Also, by obeying Poole–Frenkel (P‐F) emission, ln(*I*/*E*) versus E^0.5^ curves (P–F plot)

$$\begin{array}{ccc} & & I_{PF,T}=\frac{qA\mu N_{C}V}{t}\;exp\left[\frac{-q\left(\phi_{T}-\sqrt{qV/\pi \varepsilon_{i}\varepsilon_{0}t}\right)}{kT}\right],\;\\ & & Slope_{PF,T}=\frac{-q\left(\phi_{T}-\sqrt{qV/\pi \varepsilon_{i}\varepsilon_{0}t}\right)}{1000k} \end{array}$$

Here, *Φ*_T_ is the trap energy level, *N*_C_ is the density of states in the conduction band, *µ* is the electronic drift mobility, *T* is the absolute temperature, and *k* is Boltzmann's constant [55].

Figure S6h demonstrates that the F‐N plot for FTJ_MFIM_ remains unchanged regardless of temperature, indicating that the current conduction mechanism in FTJ_MFIM_ is primarily governed by F–N tunneling. This trend is further supported by the quantitative extraction of the effective barrier height from the F–N plots (Figure S6h), yielding a *Φ*_B_ range of 0.73–0.78 eV. In contrast, Figure S6f reveals a temperature‐sensitive variation in the F–N plot for FTJ_MFIS_, suggesting that resistive switching driven by oxygen vacancy redistribution is the dominant conduction mechanism in FTJ_MFIS_. Accordingly, the *Φ*_B_ values extracted from the F–N slopes fall in the range of 0.43–0.48 eV (Figure S6f). Furthermore, P–F emission analyses (ln(*I*/*E*) versus *E* ^1/2^) in Figure S6i,k further distinguish the two stacks: for FTJ_MFIS_, the P–F plots are linear at each temperature, evidencing field‐assisted trap emission; in FTJ_MFIM_, the P–F representation does not yield consistent linearity or a temperature dependence, in line with F–N dominated transport. Building on these P–F trends, the P–F plots (ln(*I*/*V×T^3^* ^/2^) versus 1000/*T*) in Figure S6j,l enable quantitative extraction of the trap energy level, yielding *Φ*_T_ = 0.524–0.596 eV for FTJ_MFIS_ and *Φ*_T_ = 0.397–0.471 eV for FTJ_MFIM_. Taken together, these results indicate that FTJ_MFIM_ achieves thermally robust memory characteristics because carrier conduction is governed by the thermally insensitive tunneling mechanism.

The MFIM structure exhibits low off‐current; however, it suffers from the drawback of a limited on/off ratio. In contrast, the MFIS structure achieves a higher on/off ratio but at the cost of a larger off‐current. To compare the operation voltage versus the TER ratio of the FTJ_MFIM_ with that of the FTJ_MFIS_, the maximum TER ratio (Max(TER ratio)) versus *V*_Max_ was plotted at a fixed read voltage of *V*_READ_ = 2 V, as shown in Figure S7. While the Max(TER ratio) of the FTJ_MFIM_ increases linearly and then becomes saturated with increasing *V*_Max_, that of the FTJ_MFIS_ increases exponentially after resistive switching. Notably, although the FTJ_MFIM_ has a smaller TER ratio at a large *V*_Max_, it has a larger TER ratio at a lower operating voltage before resistive switching owing to the reduced off‐current. For the implementation of low‐power large‐scale arrays, it is crucial to reduce the off‐current while maintaining or enhancing the on/off ratio to secure sensing margin. Additionally, from a resistance perspective, the integration of metal lines becomes essential in large‐area arrays for minimizing the voltage drop and RC delay. Therefore, the MFIM structure, employing a metal bottom electrode instead of silicon, was adopted, and strategies to enhance the TER ratio of FTJ_MFIM_ are suggested for stable low‐power large‐scale memory operations.

### 2.3 Design Guidelines of FTJ_MFIM_ for TER Ratio Enhancement

#### 2.3.1 Interlayer Engineering

**FIGURE 2:**
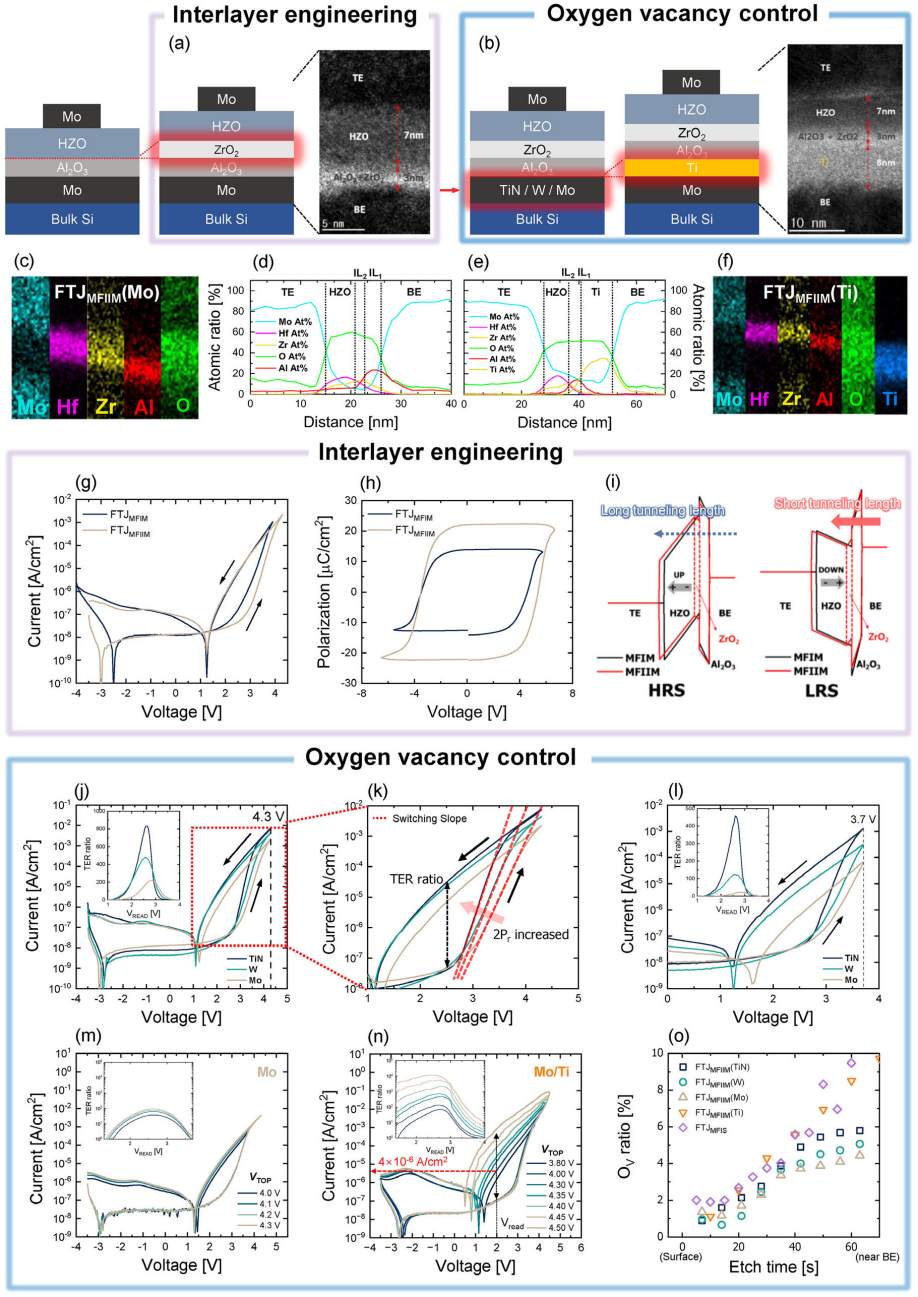
(a,b) Cross‐sectional TEM images of FTJ_MFIIM_s with inserted ZrO_2_ (1 nm) and Al_2_O_3_ (2 nm) interlayers. Especially, FTJ_MFIIM_(Ti) was fabricated by inserting a Ti metal between the interlayer and the Mo bottom electrode to enhance oxygen scavenging. (c–f) EDS elemental mappings and depth profiles for FTJ_MFIIM_(Mo) and FTJ_MFIIM_(Ti), confirming the spatial distribution of oxygen concentration. (g) *I–V* and (h) *P–V* curves of FTJ_MFIM_ and FTJ_MFIIM_ showing that ZrO_2_ insertion shifts the *V*_FN_ and enhances 2*P*_r_, leading to an increased TER ratio. (i) Energy band diagrams of FTJ_MFIIM_ illustrate tunneling paths in the HRS and LRS, highlighting the role of the ZrO_2_ interlayer in modulating barrier asymmetry. (j,k) *I–V* curves of FTJ_MFIIM_s with various bottom electrodes show different slope steepness in switching depending on *P*_r_; inset indicates improved TER ratio in FTJ_MFIIM_(TiN), indicating correlation between *P*_r_ and bottom electrode oxygen scavenging strength. (l) *I–V* curves of FTJ_MFIIM_s measured at lower *V*_Max_ = 3.7 V to more clearly distinguish TER differences among bottom electrodes; inset similarly emphasizes the enhanced contrast. *I–V* curves of (m) FTJ_MFIIM_(Mo) and (n) FTJ_MFIIM_(Ti); only the Ti‐inserted FTJ shows a second abrupt current increase above 4.3 V due to resistive switching, achieving TER > 10^4^. (o) Oxygen vacancy ratios are the XPS analysis extracted for FTJ_MFIIM_s with different bottom electrodes, showing V_O_‐rich behavior in FTJ_MFIIM_(Ti), explaining their hybrid switching characteristics. Both PUND (100 kHz triangular pulses) and DC *I–V* measurements were conducted at RT, where the DC sweeps were carried out with Δ*V* = 50 mV and *I*_comp_ = 1 mA.

To enhance the TER ratio, a ZrO_2_ of 1 nm was introduced as a second interlayer between the HZO and Al_2_O_3_ layers. Figure 2a shows cross‐sectional TEM image of the Mo/Hf_x_Zr_1‐x_O/ZrO_2_/Al_2_O_3_/Mo stack. FTJs with a metal‐FE‐IL_2_ (ZrO_2_)–IL_1_ (Al_2_O_3_)–metal stack (FTJ_MFIIM_) were fabricated using the same fabrication process as the FTJ_MFIM_, except for ZrO_2_ IL insertion. ZrO_2_ was also deposited by thermal atomic layer deposition (ALD) using TEMA‐Zr as the precursor and O_3_ as the reactant. As shown in Figure 2c,d, the distribution of elements was validated through EDS atomic mappings and line scans for FTJ_MFIIM_, respectively.

Figure 2g illustrates the hysteretic *I*_T_ curves of the FTJ_MFIM_ and FTJ_MFIIM_. With ZrO_2_ insertion, the *V*_Max_ at which tunneling starts to occur (*V*_th,1_) in the HRS is positively shifted (from 2 to 2.8 V) with a negligible change in the LRS, leading to an increasing TER ratio. Through energy‐band diagram analysis depending on the polarization status of the FE layer, the origin of the shifted *I*_T_ curve was investigated. Figure 2i shows the energy band diagrams of FTJ_MFIIM_ in the HRS and LRS, when *V*_READ,_ which does not affect the polarization state, was applied to the top electrode. For the HRS with a polarization‐up state (HRS in Figure 2i), electrons should move from the bottom electrode (BE) to the top electrode (TE) through all of the IL and FE layers by tunneling, indicating that only TAT is allowed and that negligible *I*_T_ can flow for both FTJ_MFIIM_ and FTJ_MFIM_ regardless of the additional ZrO_2_ tunneling barrier. However, when *V*_Max_ increases and reaches the threshold for polarization switching (∼*V*_th,1_), the FE becomes polarized down (LRS in Figure 2i), and *I*_T_ starts to increase exponentially because *I*_T_ is dominated by the F‐N tunneling through the IL. Here, it should be noted that the increasing amount of *I*_T_ and its slope are affected by polarization switching, which adjusts the tunneling length for the F‐N tunneling. Previous studies have reported that ZrO_2_ insertion underneath HZO enhances polarization switching by accelerating the crystallization and increasing the ferroelectric o‐phase of HZO [67]. Owing to the enhanced *P*_r_ by the ZrO_2_ IL insertion in the FTJ_MFIIM_ (Figure 2h), the FTJ_MFIIM_ has a similar LRS to the FTJ_MFIM_ despite the shifted *I*_T_ and thicker tunneling length. Therefore, a larger TER ratio can be obtained by the combination of a positive *I*_T_ shift induced by the increasing *V*_Max_ required for polarization switching and *P*_r_ enhancement, which are caused by the introduction of ZrO_2_, although the operation voltage (*V*_Max_) is slightly increased.

#### 2.3.2 Oxygen Vacancy Control Inside Ferroelectric Material

In the second method, to enhance the TER ratio, Mo at the BE was replaced with various metals to adjust the V_O_ concentration inside the HZO films via oxygen scavenging effects because it is well known that V_O_s affect the formation of ferroelectricity in HfO_2_‐based FE (Figure 2b) [68, 69]. In this study, we selected Ti, TiN, W, and Mo as BE metals of FTJ_MFIIM_s, where Ti was inserted between the IL and Mo. Figure 2b presents a cross‐sectional TEM image of the Mo/Hf_x_Zr_1‐x_O/ZrO_2_/Al_2_O_3_/Ti/Mo stack (FTJ_MFIIM_(Ti)). Furthermore, the EDS atomic mappings and line scans analysis for FTJ_MFIIM_(Ti) provide evidence for the formation of a TiO_x_ thin film, which functions as an oxygen scavenging layer (Figure 2e,f). The hysteretic *I*_T_ curves in Figure 2j confirm that the slope of polarization switching (Figure S4b) directly impacts the TER ratio in FTJ_MFIM_s. This observation indicates that enhancing *P*_r_ leads to an increase in the TER ratio as the slope trend aligns with the *P*_r_ values shown in Figure 2k. This effect was particularly pronounced during low‐voltage operation, as illustrated in Figure 2l. FTJ_MFIIM_ with TiN at the BE (FTJ_MFIIM_(TiN)) almost reached the maximum TER ratio, whereas FTJ_MFIIM_(W) and FTJ_MFIIM_(Mo) only reached 25% and 10% of the maximum TER ratio at *V*_Max_ = 3.7 V, respectively (Inset of Figure 2l). This discrepancy is attributed to the lower slope steepness of polarization switching, constrained by their respective *P*_r_ values (Figure S8a). As a result, *V*_Max_ could be reduced with a larger and steeper *I*_T_ increase without compromising the memory window. The insets of Figure 2j,I confirm that a high TER ratio is achievable at a reduced *V*_Max_, enabled by enhanced *P*_r_. Note that the FTJ_MFIIM_s with Mo, W, and TiN were switched only by polarization switching without resistive switching. As shown in Figure 2m, FTJ_MFIIM_(Mo) saturated without further *I*_T_ increase after *V*_Max_ = 4.3 V. However, the FTJ_MFIIM_ with Ti/Mo at the BE (FTJ_MFIIM_(Ti)) showed a second *I*_T_ increase by resistive switching at a high *V*_Max_ of over 4.3 V in Figure 2n, leading to > 10^4^ TER ratio. The TER ratio in the insets of Figure 2m,n reveals a clear difference in the switching mechanism between FTJ_MFIIM_(Mo) and FTJ_MFIIM_(Ti). This behavior is indicative of hybrid switching, where ferroelectric polarization switching and resistive switching coexist and synergistically influence the overall TER enhancement. In the case of FTJ_MFIIM_(Ti), the first abrupt increase in current is governed by polarization switching, while the second rise results from oxygen vacancy redistribution under high electric field, confirming the dual‐switching property. To clarify whether the second current step in FTJ_MFIIM_(Ti) originates from a bulk resistive switching mechanism or from interface/interlayer‐related modulation, we conducted polarity‐dependent DC voltage sweep measurements on 5 cells (Figure S9). If the second current step were governed by a bulk switching process, a comparable TER behavior would be expected under both voltage polarities. However, while the overall hysteresis shape and the HRS/LRS levels remained comparable within device‐to‐device (D2D) variation, a discernible TER contrast was obtained only when the polarization was oriented in the downward direction. Under the opposite polarity, no meaningful TER modulation was observed at the read voltage. These observations are consistent with interface/interlayer‐driven barrier modulation rather than a polarity switching, supporting the hybrid switching in Figure 2n. Building the hybrid switching behavior in Figure 2n, we evaluated controllability and a safe operating window via an incremental step‐stress on n = 16 of FTJ_MFIIM_(Ti). Double‐sweep *I–V* curves were recorded and two thresholds (V_th,1_ and V_th,2_) were extracted in Figure S10. Polarization switching begins at *V*_th,1_ ≈ 2.7 V, and a distinct second increasing appears once *V*_Max_ exceeds *V*_th,2_ ≈ 4.2 V. The compiled distributions yield a compact *V*_th_ map that partitions three regimes—polarization switching, hybrid switching, and risk—thereby visualizing a practical operating window (Figure S10b). At *V*_READ_ = 2 V, the current gain defined as log_10_(*I*_LRS_/*I*_HRS_) increases monotonically with *V*_Max_ (Figure S10c).

To elucidate the correlation between V_O_, *P*_r_, and resistive switching, XRD and XPS analyses were performed. Figure S11b presents the *P*_r_ values calculated from the *P–V* curves shown in Figure S8a, along with the relative fractions of the o‐, t‐, and m‐phases derived from the deconvoluted XRD peaks in Figure S11a. Detailed deconvolution of the XRD peaks in the 2θ range of 27° to 34° is provided in Figure S12a–d. Figure 2o illustrates the V_O_ ratios for FTJ_MFIIM_s with Mo, W, TiN, and Ti/Mo extracted from the XPS depth profiles in Figure S12e–h and compares them with those of FTJ_MFIS_. As expected, the o‐phase ratios decrease sequentially in the order of FTJ_MFIIM_(Ti), FTJ_MFIIM_(TiN), FTJ_MFIIM_(W), and FTJ_MFIIM_(Mo), consistent with the *P*_r_ enhancement trends observed in Figure S11b. In addition to V_O_ ratio, electrode‐induced factors such as differences in work function or polarity of oxygen vacancies could influence leakage current levels and breakdown characteristics. To examine this possibility, we compared FTJ_MFIIM_(TiN), FTJ_MFIIM_(W), and FTJ_MFIIM_(Mo), as shown in Figure S8b,c. The leakage current levels, the onset voltages for *V*_FN_, and the breakdown voltages are all comparable, and the corresponding changes in tunneling current are negligible relative to the effects of ferroelectric polarization and the resulting TER modulation. Collectively, these results indicate that differences in electrode work function are not the primary determinant of device behavior. Rather, the dominant factor among electrodes is the oxygen vacancy concentration within HZO. Additionally, the oxygen vacancy distribution within HZO near the interface showed that FTJ_MFIIM_(Ti) had an V_O_ concentration comparable to that of FTJ_MFIS_ (Figure 2o). Thus, it was verified that the V_O_ concentration plays a critical role in the formation of ferroelectricity in HZO, while excessive V_O_ levels can trigger resistive switching in FTJ_MFIIM_s through a mechanism similar to that in FTJ_MFIS_—namely, barrier lowering caused by V_O_ accumulation. This dual contribution of polarization and resistive switching reaffirms the hybrid switching characteristics of FTJ_MFIIM_(Ti), especially under high electric field stress conditions.

To further verify the mechanism separation in FTJ_MFIIM_(Ti), we performed temperature‐dependent P–F and F–N analyses at 25, 45, 65, 85°C in Figure S13. In the polarization‐switching region (*V*_READ_ ≈ 3.5 to 3.8 V, Δ*V*_READ_ = 100 mV), the P–F plots (ln(*I/E*) versus *E* ^0.5^) in the Figure S13d are non‐linear at all temperatures, while the F–N plots (ln(*I/E* ^2^) versus 1/*E*) show a nearly temperature‐invariant slope, indicating that transport is consistent with polarization switching induced F–N tunneling rather than P–F emission. Consistently, fitting the polarization switching regime in Figure S13a yields an effective barrier height of *Φ*_B_ = 1.21–1.26 eV. By contrast, in the hybrid switching region (*V*_READ_ ≈ 4.3 to 4.5 V, Δ*V*_READ_ = 50 mV), the P–F plots in the Figure S13e become linear with a temperature dependence, and the F–N slope also becomes temperature‐dependent, evidencing V_O_‐assisted, field‐enhanced trap emission superimposed on F–N tunneling. To quantify the trap‐related contribution, we further analyze the temperature dependence using ln(*I*/(*V×T* ^3/2^)) versus 1000/*T* (Figure S13b,c), from which the extracted trap energy level falls in the ranges of *Φ*_T_ = 0.336–0.417 and 0.324–0.381 eV depending on the applied read‐voltage. Taken together, these diagnostics support the coexistence of polarization switching and V_O_‐driven resistive switching in FTJ_MFIIM_(Ti).

Figure S11c shows a comparison of the TER ratios among the devices, demonstrating that the FTJ_MFIIM_s have a larger TER ratio than the FTJ_MFIS_ despite the lower *V*_Max_ owing to the ZrO_2_ IL insertion and V_O_ adjustment. However, it should be noted that FTJ_MFIIM_(Ti) has a comparable off‐current to other FTJ_MFIIM_ devices although FTJ_MFIIM_(Ti) has high V_O_ concentrations near the interface between HZO and IL. This discrepancy can be understood by the energy bands of FTJ_MFIIM_(Ti). To provide a unified comparison among all FTJs investigated in this work, the applied top‐electrode bias was converted to the effective ferroelectric field (*E*_FE_) across the HZO layer using a series‐capacitance model. The detailed material parameters employed for this conversion, such as the work function (*Φ*), electron affinity (*χ*), dielectric constant (*κ*), and physical thickness of each layer, are summarized in Table S1 [70, 71, 72, 73, 74, 75, 76, 77, 78, 79, 80, 81, 82, 83, 84, 85]. The corresponding *V*_FE_–*E*_FE_ mapping and regime interpretation are illustrated in Figure S14, which annotates thresholds of the polarization switching, the saturation region, and the hybrid switching for each stack. Based on this conversion, the read operation (*V*_READ_ = 2 V) corresponds to *E*_FE_ ≈ 1.1 MV/cm, the polarization switching occurs near *E*_FE_ ≈ 1.4–1.5 MV/cm, and the hybrid switching emerges above *E*_FE_ ≈ 2.3–2.4 MV/cm. These field‐based analyses clarify the physical boundaries between the distinct switching regimes and explain the apparent saturation behavior and asymmetry observed in Figures 1 and 2, providing a consistent framework for the subsequent array‐level discussion. In addition, Figure S15 compares the program (PGM) and erase (ERS) band profiles, highlighting how the built‐in potential distribution differs across HZO and the ILs under identical voltage conditions.

To assess the feasibility of FTJ_MFIIM_(Ti) for NVM applications, its switching speed and reliability, including endurance and retention characteristics, were investigated at *V*_READ_ = 2 V (Figure S16). Figure S16b demonstrates that the polarization switching and resistive switching are clearly separated based on ∼4 × 10^−6^ A/cm^2^ at low program voltage, indicating that a longer program time is required for resistive switching due to slower V_O_ response compared to polarization switching. Notably, this separation is consistent with the hysteretic *I*_T_–*V* curves of Figure 2n, where the *I*_T_ starts to increase by resistive switching from 4 × 10^−6^ A/cm^2^. However, as the program voltage increases, the two switching mechanisms converge by the accelerated V_O_ movement under a stronger electric field across HZO, suggesting that FTJ_MFIIM_(Ti) achieves a switching speed comparable to other FTJ_MFIIM_s despite its larger TER ratio.

Endurance and the retention characteristics of the FTJ_MFIIM_(Ti) and the FTJ_MFIIM_(Mo) were measured and compared in Figure S16e–h, based on the switching properties of the FTJs shown in Figure 2m,n. The *I*_T_ evolutions of program and erase states were extracted with respect to the number of cycling (Figure S16e,f). It is observed that all the FTJs exceed 10^8^ cycles. The high current level shows a slow degradation, and the low current level similarly shows an increase; yet, the current separation retains a wide margin until 10^8^ cycles. After the cycling stress, the retention properties were monitored at 25°C as a function of time. Figure S16h indicates that *I*_T_s slightly decrease by V_O_ redistribution within few seconds for FTJ_MFIIM_(Ti), and then it maintains depolarization‐dominated retention properties. In contrast, for FTJs without resistive switching, *I*_T_ change is negligible without an initial sharp *I*_T_ decrease in Figure S16g,h. For both the FTJs, the LRS and HRS remain stably separated till 10^4^ s, although the current difference is slightly reduced by the depolarization of HZO in FTJ_MFIIM_(Ti), which demonstrates the feasibility of NVM operations.

### 2.4 Large‐Scale FTJ_MFIIM_ Array and Its Neuromorphic Applications

#### 2.4.1 Large‐Scale FTJ Array Operations

**FIGURE 3:**
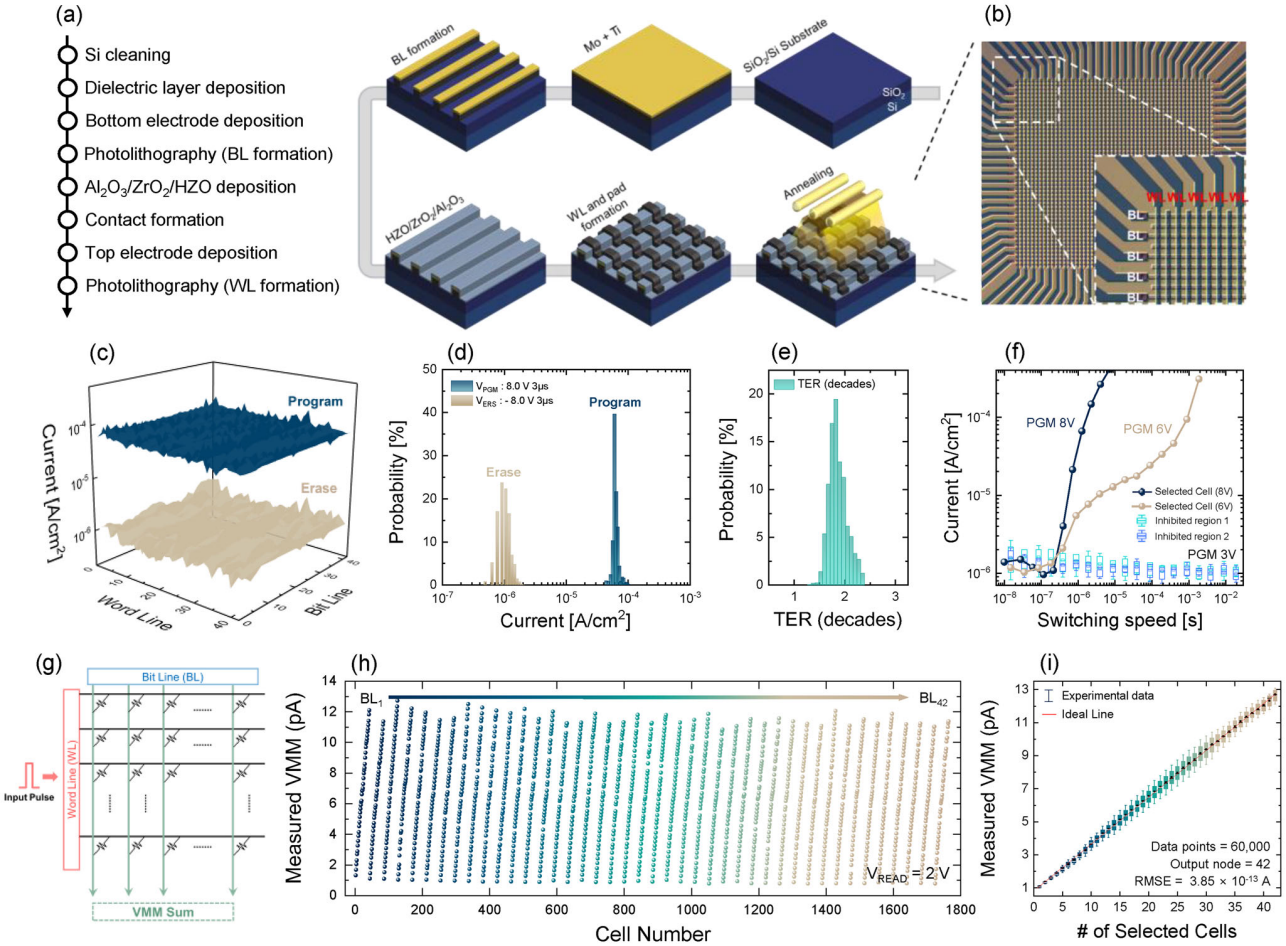
(a) Fabrication process and (b) corresponding optical image of the 42 × 42 FTJ_MFIIM_(Ti) array, showing the BL/WL patterning and array layout. (c) 3D *I–V* plot of program/erase states measured across the full array show uniform current levels in both states. (d) Program and erase currents distribution of FTJ_MFIIM_(Ti) array extracted at *V*_READ_  =  2 V after ± 8 V/3 µs pulse condition. (e) TER (decades) distribution of FTJ_MFIIM_(Ti) array, showing a narrow spread with an average memory window of approximately 2 orders of magnitude. (f) Switching speed of FTJ_MFIIM_(Ti) devices in the array as a function of pulse width, showing fast ferroelectric switching and successful inhibition at *V*_PGM_/2 without unintended programming. (g) VMM operation scheme of the FTJ_MFIIM_(Ti) synaptic array, illustrating input through WLs and output integration through BLs for efficient in‐memory computation. (h) Measured VMM results across 42 WLs and 42 BLs under *V*_READ_ = 2 V, demonstrating uniform and linear current accumulation across all 1764 cells. (i) VMM outputs depending on the number of selected cells show excellent linearity and accumulation accuracy over 60 000 data points (RMSE = 0.385 pA). All electrical properties were measured at RT with *I*_comp_ = 1 mA.

In this study, a large‐scale FTJ crossbar array of FTJ_MFIIM_(Ti) was fabricated to validate reliable operation for neuromorphic computing applications. Figure 3a,b shows the fabrication process and optical microscope image of the FTJ crossbar array, respectively. The FTJ crossbar array comprises 42 word‐lines (WLs) and 42 bit‐lines (BLs), forming a 42 × 42 matrix with a total of 1764 cells, each occupying an area of 100 µm^2^. The WLs and BLs serve as horizontal and vertical metal lines, respectively, enabling individual access to each cell in the array. Program and erase operations were performed on each cell using ±8 V pulses with a 3 µs width to ensure complete polarization switching. Read operations were conducted at a read voltage of 2 V, effectively distinguishing between the low resistance state (LRS, 10^−4^ A/cm^2^) and high resistance state (HRS, 10^−6^ A/cm^2^) without disturbing the cell states. Figure 3c presents a 3D *I*_T_ map of the entire FTJ crossbar array, highlighting the uniformity of program and erase states across the array. Figure 3d illustrates the distribution of cells in their program and erase states, showing minimal cell‐to‐cell (C2C) variation—an essential characteristic for high‐density arrays. As shown in Figure 3e, the TER (decades) distribution across the crossbar array demonstrated approximately two orders of magnitude, providing sufficient margin to represent multi‐level conductance states for updating synaptic weights.

For the FTJ crossbar array to operate reliably as a NVM device in neuromorphic applications, it is essential that the selected cells exhibit independent operations. To prevent disturbance of unselected neighboring cells during program or erase operations on the selected cell, an inhibition method was implemented [18]. During programming or erasing operations, the WL connected to the selected cell was supplied with the full program or erase voltage (*V*_PGM_ and *V*_ERS_), while the BL connected to the selected cell was grounded (0 V). Simultaneously, half of the programming or erasing voltage was applied to unselected WLs and BLs to suppress unintended polarization switching in unselected cells (Figure S17). Cells in the inhibited regions received voltages insufficient to induce polarization switching, ensuring they remained in the HRS. As shown in Figure 3f, the selected cell successfully switched to the LRS under the applied conditions, while unselected cells in inhibited region were unaffected. By incorporating inhibition operations into the FTJ crossbar array, each cell can be independently selected without interference from neighboring cells. This capability for independent switching is essential for neuromorphic applications, where precise control of synaptic weights is critical for achieving accurate neural network performance.

To assess the parallel computational capabilities of the neural network, the vector matrix multiplication (VMM) operation was validated. Figure 3g provides a schematic representation of the VMM operation for neuromorphic computing. For these operations to remain reliable, particularly as the FTJ crossbar array scales up, it is critical for the array to exhibit self‐rectifying characteristics [86]. Figure 3h,i presents the VMM operations performed at the initial states of selected cells across all BLs, with all operations conducted at a read voltage *V*_READ_ = 2 V. The currents measured at each BL confirm that reliable VMM operations are achievable, exhibiting linearity proportional to the number of selected cells in each BL. To suppress undesired leakage and maintain the integrity of current accumulation during VMM, two complementary strategies were implemented. First, a differential‐synapse configuration using two FTJs per synapse (*G* ^+^ and *G* ^−^) defines the synaptic output as the current difference (*G* ^+^–*G* ^−^), effectively canceling common leakage components. Second, a bias‐inhibition scheme was applied such that all unselected cells were held at 0 V while the selected cells were read at *V*_READ_ = 2 V, minimizing sneak‐path currents. As shown in Figure S18a,b, the current remains essentially unchanged at 0 V even for LRS devices, and the device‐to‐device variations confirm robust separation between HRS and LRS at 2 V. Particularly, Figure 3i highlights results from random addressing of the 42 WLs, with current collected from a single BL. The dataset comprises 60 000 data points. For cases where the number of selected WLs was fewer than 12 or greater than 30, 1000 addresses were randomly selected and measured for each scenario to ensure the accuracy of the results. The observed variations and distributions confirm that the sum of currents from the randomly selected cells matches the calculated VMM current, closely following the ideal linear trend with a root‐mean‐square error (RMSE) of 0.385 pA. These findings underscore the capability of the FTJ crossbar array to perform accurate VMM operations, even under random cell selection, demonstrating its potential for reliable execution in hardware‐based neuromorphic computing applications.

#### 2.4.2 In‐Memory Vision Transformer System Using FTJ Array

**FIGURE 4:**
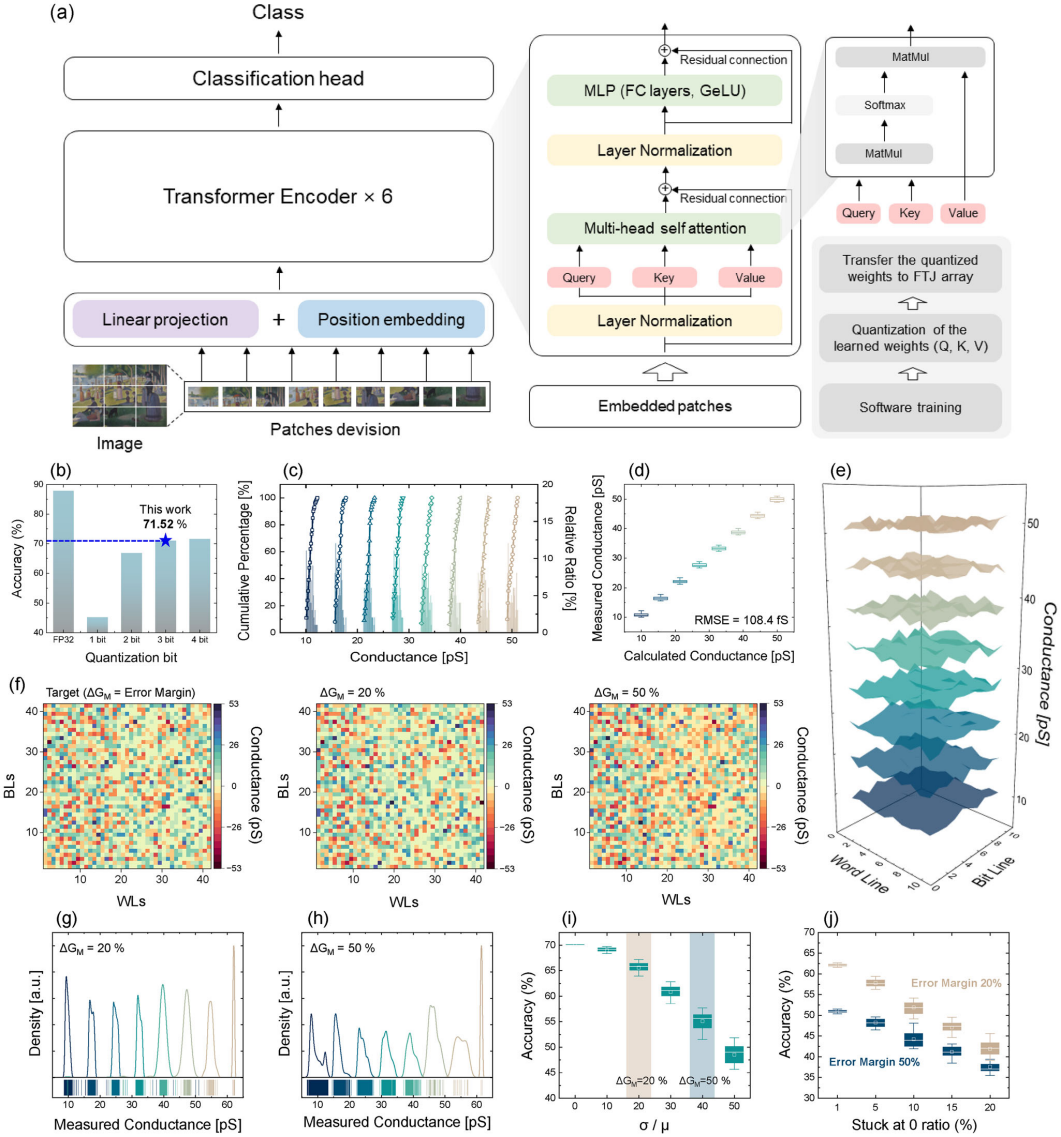
(a) Off‐chip based ViT system architecture based on an FTJ array. (b) ViT classification accuracy versus weight quantization bit width. Each weight is quantized into 2*^N^*−1discrete levels, where *N* is the bit precision. (c) Cumulative conductance distribution extracted from experimentally tuned states, showing clear separation among 8 levels. (d) Box plot and (e) 3D conductance map of 8 conductance levels measured across 100 FTJ cells. The results demonstrate reliable and uniform multi‐level programming, confirming precise cell‐level control within the FTJ array. (RMSE = 108.4 fS). (f) Comparison between the target and transferred weights in the synaptic 42 × 42 FTJ array under different tuning conditions. The weights are fine‐tuned with 20% and 50% tuning margins and subsequently quantized to 3 bits. Weight distributions based on conductance error in 8‐level quantization for (g) ±20%, and (h) ±50% tuning margin. (i) Effect of device variation (σ/µ) on classification accuracy. Shaded areas represent results within the tuning margins, highlighting the tolerance‐dependent impact of device variation. (j) Impact of stuck‐at‐0 cell ratio on classification accuracy under different tuning margins. All electrical properties were measured at RT with *I*_comp_ = 1 mA.

In this work, we propose an in‐memory ViT system that incorporates an FTJ array as an artificial synaptic device optimized for large‐scale vision models. ViTs are characterized by high power consumption and prolonged computation times due to the substantial number of weight parameters and complex computation. Transitioning from conventional von Neumann architecture to in‐memory ViT systems leveraging FTJ arrays offers a promising solution for reducing resource consumption. The proposed in‐memory ViT system architecture based on a synaptic FTJ array is depicted in Figure 4a. Unlike convolutional neural networks (CNNs), ViTs lack an inductive (learning) bias but employ self‐attention mechanisms commonly used in natural language processing (NLP). In ViT systems, images are processed by dividing them into patches, with each patch traversing multiple layers and being handled with different weights. After splitting the image into patches and embedding them, ViT adds positional embeddings and passes the patch tokens through multiple Transformer encoder layers, where self‐attention and multi‐layer perceptron (MLP) layers refine the representation. Each input patch is linearly projected into query, key, and value vectors by the self‐attention mechanism of ViT. Here, the dot product of the query and key determines the attention weights, and the weighted sum over values enables context‐aware feature refinement by capturing inter‐patch relationships. In this work, the optimized query, key, and value weights obtained from software training were transferred onto the fabricated synaptic FTJ array to enable hardware‐level execution. Considering the inherent trade‐off between the enhanced TER achievable in the V_O_‐redistribution‐driven resistive switching regime and degraded retention/endurance, we intentionally focus on the more stable polarization switching for synaptic operation. In an off‐chip training framework, any retention‐related state relaxation can be mitigated by occasional refresh/reprogramming of the software‐trained weights.

A ViT model was trained on the CIFAR‐10 dataset with hardware‐aware constraints, including input binarization and post‐training quantization. The input images were divided into non‐overlapping patches of size 16 × 16, and each patch was embedded into a 42‐dimensional vector, aligned with the hardware constraints of the synaptic FTJ array. Detailed simulation settings and training procedures are described in the Methods section. Figure 4b shows the post‐training quantization results as a function of the number of conductance states. To evaluate the impact of hardware non‐idealities during weight mapping, quantization was selectively applied to the query, key, and value weights only. The quantization bits (*N*) correspond to the number of conductance states of the FTJs, resulting in a total of 2*^N^*‐1 distinct levels for representing signed weights. A significant degradation in accuracy of approximately 43% was observed with 1‐bit quantization due to severe information loss. However, the accuracy recovered as the quantization bits increased, reflecting reduced information loss. For instance, 3‐bit and 4‐bit quantization resulted in accuracy drops of 16.5% and 16.0%, respectively, suggesting that the accuracy begins to saturate beyond 3 bits. Figure S19b presents the accuracy trends with respect to quantization bits, based on the results from 10 repeated independent experiments. Considering the tuning margin of the FTJs, 3‐bit weight quantization was performed in the FTJ‐based ViT system.

Therefore, the ability to accurately tune and learn diverse synaptic weights is critical for performance optimization. The multi‐level conductance characteristics of the FTJ synapses play a pivotal role in enabling precise, continuous updates and transfer of trained weights, enhancing the performance of neural networks. Figure 4c provides the C2C variation and cumulative distribution of the implemented 8‐level conductance states, underscoring the FTJ array's consistency and reliability. Figure 4d,e illustrates the variability of data for eight distinct conductance states in the weight range of 10–50 pS across 100 cells (10 WLs × 10 BLs = 100 cells), presented via a 3D map and box plots. Each state was implemented through accurately calculated and measured conductance, demonstrating the FTJ array's capability to stably represent multiple weight states. To quantitatively assess this capability, the RMSE between the target and measured conductance values was computed across all levels, resulting in a low error of 108.4 fS, indicative of precise synaptic programming.

In neuromorphic computing, long‐term potentiation (LTP) and long‐term depression (LTD) characteristics are foundational mechanisms for learning and memory formation in biological neurons, serving as key principles for synaptic weight adjustment in artificial systems. These characteristics visually represent changes in synaptic weights, offering critical indicators for assessing linearity and repeatability in weight updates. Figure S20 presents 3‐bit LTP/LTD curves, highlighting the FTJ array's ability to learn and adjust multi‐level conductance states, as well as its cycling endurance. Weight updating was performed 2 × 10^3^ times within the conductance range of 10–50 pS, confirming stable LTP/LTD characteristics during repetitive learning cycles. All measured multiple weight states of FTJ crossbar array were obtained using the tuning method algorithm described in Figure S21 and measured at *V*_READ_  =  2 V. The robust and stable control of conductance values in the FTJ crossbar array confirms its effectiveness in performing reliable weight updates, a crucial requirement for neural network learning in ViT systems.

Based on the synaptic characteristics of the fabricated FTJs, the software‐trained optimal query, key, and value weights were transferred to the hardware. Due to inherent device variability, exact weight values obtained from off‐chip training are difficult to reproduce in hardware, necessitating the use of a tuning error margin during weight transfer. The extent of the tuning error margin plays a critical role in maintaining functional accuracy, as excessively narrow margins may lead to programming failures, while excessively wide margins can degrade model performance due to weight distortion. This is particularly important in FTJs, where the conductance states are closely spaced, making the system more vulnerable to tuning inaccuracies. The effect of tuning precision on the system performance was evaluated by applying two distinct error margins during the weight transfer process, 20% and 50%. Considering possible disturbances during device programming, synaptic weights were transferred through multiple iterations to enhance tuning reliability. The detailed fine‐tuning procedure is described in the Methods section.

To implement the in‐memory ViT system, the synaptic weights corresponding to the pretrained query, key, and value components were transferred to the synaptic FTJ array. In the in‐memory ViT architecture, each synaptic weight is represented by two synaptic FTJs, *G* ^+^ and *G* ^−^, to encode negative weight values through differential conductance. Figure S22 presents a comparison between the simulated and experimentally programmed *G* ^+^ and *G* ^−^ conductance values for the query, key, and value weights, as transferred to the FTJ array. This close agreement confirms the effectiveness of the weight mapping process and the feasibility of accurate synaptic programming in the FTJ‐based in‐memory ViT system. Figure 4f presents the experimentally programmed query weights under two tuning margins (20% and 50%), compared with the corresponding software‐derived target values. Figure 4g,h illustrates the distribution of the programmed weights under each tuning margin, reflecting the variability introduced during the transfer process, while Figure S23 presents the number of programming pulses required to reach each conductance state under the same margin conditions. These results demonstrate a trade‐off between tuning precision and programming effort: a smaller margin enables more accurate weight transfer with tightly clustered conductance values but requires significantly more pulses to reach the target state, thereby increasing the overall time and energy consumption during the weight mapping process. In contrast, a larger margin, such as 50% reduces the number of required pulses and speeds up the programming process, but at the cost of reduced accuracy due to broader and more overlapping conductance distributions.

To investigate the tuning margin–induced performance degradation, the experimentally measured weight distributions were incorporated into simulations to evaluate their impact on system performance. Figure 4i shows the effect of tuning margin (σ/µ, standard deviation over mean) on the inference accuracy in off‐chip learning. The simulation was conducted by gradually increasing Gaussian noise on the quantized weights, and 20 independent inference tests were performed under each noise condition. The shaded regions correspond to the σ/µ ranges experimentally observed under 20% and 50% tuning margins. As the variation increases, the accuracy significantly degrades by about 5% to 10% and about 15% to 20% for the 20% and 50% margin, respectively. This degradation becomes more pronounced with larger tuning margins, especially in FTJs, where the narrow spacing between conductance states makes the system more sensitive to programming imprecision and external noise. Furthermore, the effect of stuck cells, a representative hardware‐level non‐ideality, was investigated to assess their influence on the inference accuracy of the in‐memory ViT system. Figure 4j shows the inference accuracy as a function of the stuck cell ratio under different tuning margin conditions. The inference accuracy decreases with an increasing stuck‐at‐off defect ratio, and this degradation is more severe under the 50% tuning margin condition. This indicates that larger tuning margins make the system more vulnerable to hardware defects such as stuck cells.

## 3 Conclusion

In this work, we systematically investigated the material, structural, and electrical optimization of FTJs to realize high‐performance hybrid‐switching memory suitable for both nonvolatile memory and neuromorphic computing applications. By engineering the stack structure—particularly through the integration of ZrO_2_ interlayers and the modulation of oxygen vacancy concentration via bottom electrode selection—we demonstrated tunable switching characteristics that allow for either pure ferroelectric, pure resistive, or hybrid‐switching behavior. FTJs employing Ti‐based bottom electrodes exhibited the highest TER ratios due to enhanced remnant polarization and resistive switching driven by V_O_ redistribution. These devices also exhibited stable endurance and retention, validating their applicability to long‐term memory operations.

Furthermore, a large‐scale FTJ array composed of optimized MFIIM stacks was fabricated and shown to operate reliably with minimal cell‐to‐cell variation. The array demonstrated multi‐level conductance tuning with minimal read disturbance, enabling its use as an artificial synaptic array for neuromorphic inference. We implemented an in‐memory ViT system utilizing this FTJ array, achieving 3‐bit weight quantization and experimentally validating the effect of tuning margins and device non‐idealities on classification accuracy. Our results confirm the feasibility of FTJ‐based hardware accelerators for in‐memory deep learning and highlight the potential of hybrid‐switching FTJs as a scalable and energy‐efficient platform for neuromorphic applications.

## 4 Methods

### 4.1 Fabrication Process of Optimized FTJs for Huge TER Ratio

#### 4.1.1 FTJ_MFIS_ and FTJ_MFIM_

Figure 1a,c shows schematics of two types of FTJs: an FTJ with metal‐ferroelectric (FE)–interlayer (IL)–Si stacks (FTJ_MFIS_) and an FTJ with metal‐FE‐IL‐metal stacks (FTJ_MFIM_). The FTJs were fabricated on a highly doped p‐type silicon substrate. Cleaning was performed for 20 min in a 70°C using an SC‐1 solution (NH_4_OH:H_2_O_2_:H_2_O = 1:1:5) to remove impurities, followed by native oxide removal using buffered oxide etchant (NH_4_F:HF = 6:1). For the FTJ_MFIS_, thermal ALD was utilized at 330°C with O_3_ as a reactant to deposit 30 cycles of SiO_2_ layer using BDEAS precursor and 71 cycles of HZO (Hf: Zr = 2:1 ratio) layer using TEMA‐Hf and TEMA‐Zr precursors. The top electrode (50 nm Mo) was deposited by DC sputtering, and a circular pattern (1.25 × 10^−4^ cm^2^) was formed using lithography and dry etching with reactive ion etching (RIE) using Cl_2_ gas. Finally, rapid thermal annealing (RTA) was performed for crystallization of the HZO films at 500°C for 30 s (400°C for ReMem device). For the FTJ_MFIM_, Mo bottom electrodes were deposited by DC sputtering onto similarly cleaned Si substrates, followed by 25 cycles of Al_2_O_3_ layer deposition using TMA precursor and identical HZO layer deposition as in the MFIS structures. The top electrode was patterned into the same shape as that of the FTJ_MFIS_, and post‐metallization annealing (PMA) was carried out at 500°C for 30 s in an N_2_ atmosphere to crystallize the HZO films.

#### 4.1.2 Unit FTJ_MFIIM_ and Large‐Scale FTJ_MFIIM_ Array

An FTJ with metal‐ferroelectric (FE)–interlayer (IL_2_)–interlayer (IL_1_)–metal stacks (FTJ_MFIIM_) was fabricated in the same fabrication process as the FTJ_MFIM_, except for the use of an added second interlayer and different metals of the bottom electrode (Figure 2a). Thin films of Al_2_O_3_ (25 cycles), ZrO_2_ (12 cycles), and HZO (71 cycles) were sequentially deposited using thermal atomic layer deposition (ALD) at 330°C. The FTJ_MFIIM_ stacks differ from the FTJ_MFIM_ stacks only by the insertion of a 1 nm ZrO_2_ layer between Al_2_O_3_ and HZO. And Mo, W, and Ti were deposited in an Ar atmosphere by DC sputter, and TiN was deposited by plasma‐enhanced ALD (PEALD). In PEALD, TiCl_4_ was used as a precursor, and NH_3_ gas was used as a reactant at 400°C. As shown in Figure 2b, FTJ_MFIIM_s were fabricated using TiN, W, Mo, and Ti as bottom electrodes. For the Ti‐based FTJ, the fabrication process was identical to that of the TiN, W, and Mo FTJs, except Ti (5 nm thick) was sequentially deposited on the Mo layer.

The FTJ crossbar array utilized the same Mo/HZO/ZrO_2_/Al_2_O_3_/Ti/Mo stack as the FTJ_MFIIM_(Ti) in Figure 2b. However, additional process steps were incorporated to enable individual and independent memory operations for each cell. Prior to the deposition of HZO, ZrO_2_, and Al_2_O_3_ thin films, photolithography was used to form the BLs on the Mo/Ti bottom electrode stack. WLs and pad metals were created through the contact hole and top electrode etching processes.

### 4.2 Electrical Measurement

Measurement of electrical characteristics: The electrical characteristics of the devices were evaluated using a comprehensive suite of measurement techniques and equipment. The DC double sweep measurements were conducted with a 4156B analyzer. For assessing the polarization of the ferroelectric layer, PUND measurements were performed using a Keithley 4200‐SCS parameter analyzer in conjunction with a 4225‐PMU ultrafast current–voltage module. This method enabled the extraction of the ferroelectric switching component in response to time‐transient triangular voltage pulses, from which the *P–V* curve was calculated. The triangular pulse frequency was set at 100 kHz. To characterize the memory properties of the FTJs, including switching speed, retention, and endurance, a Keysight B1500A semiconductor device analyzer equipped with SPGU and WGFMU modules was employed. The electrical characteristics of FTJs in the array and the operation of various schemes were evaluated using a probe station and a custom‐designed probe card. The measurement system incorporated a Keysight 4156B semiconductor parameter analyzer and a Keysight 81110A pulse generator as signal sources. These signals were routed to the probe cards via a Keysight E5250A switching matrix, enabling precise control and measurement of individual devices within the array (Figure S24).

XPS and XRD analyses: Stacks were prepared for material analysis. After proceeding to RTA in the same manner as for the primary samples, the top electrodes were removed by wet etching (SC‐1 solution, NH_4_OH:H_2_O_2_:H_2_O = 1:8:64, 45°C, 10 s). The prepared samples were analyzed using XPS (Thermo Fisher Scientific, NEXSA G2) and GIXRD (X'pert Pro MRD, incident angle of 0.5°).

### 4.3 TCAD Simulation

To determine the FTJ energy band diagrams, 2 nm Al_2_O_3_ for the interlayer, 1 nm ZrO_2_ for the second interlayer, 6 nm HZO for the ferroelectric layer, and 10 nm Mo for the bottom electrode were selected. The dielectric constant of the HZO was set to 25. In addition, a 3 nm TiO_2_ layer (dielectric constant ≈ 100, electron affinity ≈ 4.3 eV) was included. All FTJ energy‐band evaluations were performed using a commercial TCAD tool (Synopsys Sentaurus). The Preisach model was applied to consider the energy band diagram along the polarization direction, which is expressed as follows:

$$P_{aux}=c\cdot P_{s}\cdot tanh\left(w\cdot \left(E\pm E_{c}\right)\right)+P_{off}$$

$$w=\frac{1}{2E_{c}}\;ln\frac{P_{s}+P_{r}}{P_{s}-P_{r}}$$

where *P*_aux_ is the auxiliary polarization, *P*_s_ is the saturation polarization, *E* is the electric field, *E*_c_ is the coercive field, and *P*_r_ is the remnant polarization.

### 4.4 Weight Tuning Method of FTJ_MFIIM_ Array for ViT

To implement and evaluate multi‐level weight states in the FTJ array, a custom LabVIEW‐based control system was developed. The measurement setup integrated a Keysight 4156B semiconductor parameter analyzer, a Keysight 81110A pulse generator, and a Keysight E5250A switching matrix, enabling independent addressing of WLs and BLs. Read operations were performed at a voltage of 2 V (*V*_READ_), which was chosen to avoid any impact on the polarization state of the FTJs. During measurements, all non‐selected cells were maintained in an inhibited state to minimize unwanted interference such as read disturbance.

To modulate the weight of a selected FTJ cell, PGM and ERS voltage pulses were applied through the corresponding WL and BL. The initial PGM/ERS pulse conditions were set to +6 V and −6 V with a pulse width of 1 µs. Fine‐tuning of the weight was performed using an Incremental Step Pulse Programming (ISPP) method, in which the pulse amplitude was gradually adjusted in 0.1 V steps. To prevent cell breakdown within the array, the maximum PGM/ERS voltage was limited to 8 V. The weight tuning process was considered complete when the read conductance (*G*_R_) was within 20% of the target conductance (*G*_T_), satisfying the condition (|*G*_R_−*G*_T_| < Δ*G*_M_). If this condition was not met, the amplitude of the applied pulses was incrementally increased or decreased and reapplied until the final weight fell within the specified error margin.

### 4.5 Learning Optimizer and Hyperparameters

To evaluate the system‐level performance of the ViT, simulations were conducted using a software‐trained ViT model, where attention and MLP weights were mapped to the corresponding synaptic devices. The model was trained on the CIFAR‐10 dataset, with the input images divided into non‐overlapping patches of size 16 × 16. To align with the hardware constraints of the synaptic device array, the input images were binarized prior to patch embedding. The ViT architecture consisted of 6 encoder blocks, each with 8 attention heads and an embedding dimension of 42, and GELU was used as the activation function. The constructed network was trained for 100 epochs using a batch size of 128, with the cross‐entropy loss function and the AdamW algorithm as shown in Figure S14a. After training, Post‐training quantization was applied after model convergence, where weights were uniformly quantized based on their minimum and maximum values to fixed‐point representations. This method allows for reduced memory footprint and hardware compatibility without requiring retraining or calibration with labeled data. The initial weights were sampled from a Gaussian distribution with a mean of 0 and a standard deviation of 0.1.

## Conflicts of Interest

The authors declare no conflict of interest.

## Supporting information




**Supporting File**: advs73905‐sup‐0001‐SuppMat.docx.

## Data Availability

The data that support the findings of this study are available from the corresponding author upon reasonable request.
