# Supplementary material for: Tunable Switching Mechanisms in HfZrO2‐Based Tunnel Junctions for High‐Performance Synaptic Arrays
Source: Adv Sci (Weinh). 2026 Feb 23;13(18):e16478. doi: 10.1002/advs.202516478 (PMC13042731; doi:10.1002/advs.202516478)
Supplement: Supplementary file 1 — Supporting File: advs73905‐sup‐0001‐SuppMat.docx. [file ADVS-13-e16478-s001.docx]

Supplementary Information

**Tunable Switching Mechanisms in HfZrO_2_-Based Tunnel Junctions for High-Performance Synaptic Arrays**

Jiwon You^1†^, Jeong-Han Kim^2†^, Minsuk Song^3†^, Been Kwak^3^, Eun Chan Park^1^, Manh-Cuong Nguyenc^4^, Wonjun Shin^5*^, Jangsaeng Kim^6, 7^*, and Daewoong Kwon^1, 2, 3^*

J. You, E. C. Park, D. Kwon

^1^Department of AI Semiconductor Engineering, Hanyang University, Seoul 04763, Republic of Korea

E-mail: dw79kwon@hanyang.ac.kr

J.-H. Kim, D. Kwon

^2^Department of Electrical Engineering, Hanyang University, Seoul 04763, Republic of Korea

M. Song, B. Kwak, D. Kwon

^3^Department of Nanoscale Semiconductor Engineering, Hanyang University, Seoul 04763, Republic of Korea

M.-C. Nguenc

^4^Department of Materials Science and Engineering 3-D Convergence Center, Inha University, Incheon 22212, Republic of Korea

W. Shin

^5^Department of Semiconductor Convergence Engineering, Sungkyunkwan University, Suwon 16419, Republic of Korea

J. Kim

^6^Department of Electronic Engineering, Sogang University, Seoul 04107, Republic of Korea

^7^Department of System Semiconductor Engineering, Sogang University, Seoul 04107, Republic of Korea

*^†^ These authors contributed equally: Jiwon You, Jeong-Han Kim, Minsuk Song*

** Corresponding author (e-mail: dw79kwon@hanyang.ac.kr, kimjs@sogang.ac.kr, swj0107@skku.edu)*

**Contents**

Supplementary Figure S1–S24, Table S1

**Supplementary Figures**

**Supplementary Figure S1.** (a) Schematic and TEM image of FTJ_MFIS_(Mo/HZO/Al_2_O_3_/p⁺-Si) using Al_2_O_3_ as an interlayer. (b) *I–V* curves (Δ*V* = 50 mV and *I*_comp_ = 1 mA at RT) and (c) TER ratio under varying *V*_TOP_​, exhibiting counterclockwise hysteresis. (d) *P-V* and *I-V* curves (100 kHz triangular pulses at RT) of FTJ_MFIS_ confirming ferroelectricity.


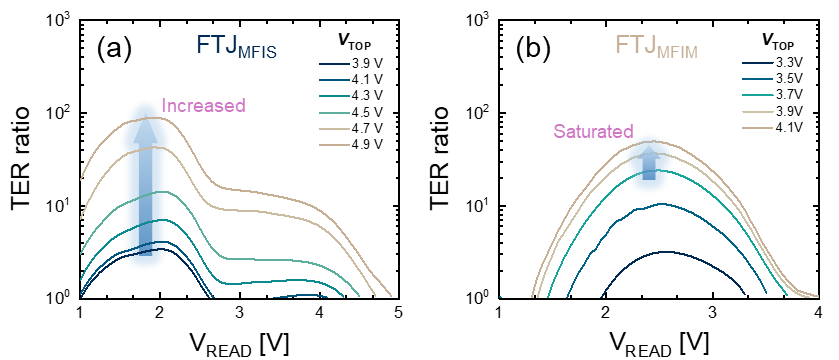


**Supplementary Figure S2.** (a, b) TER ratio for (a) FTJ_MFIS_ and (b) FTJ_MFIM_ under varying *V*_TOP_ conditions. FTJ_MFIS_ shows a continuous TER increase with *V*_TOP_, while FTJ_MFIM_ exhibits early saturation due to the absence of resistive switching.

**Supplementary Figure S3.** (a) Schematic of the ReMem structure annealed at 400 °C, a temperature insufficient to induce ferroelectricity in HZO. (b, c) *P-V* and *I-V* curves of ReMem showing negligible ferroelectric response, while MW appears at high *V*_TOP_ owing to resistive switching. (d–f) XRD patterns of FTJ_MFIS_, FTJ_MFIM_, and ReMem before and after annealing, revealing the presence of ferroelectric orthorhombic phase in FTJ_MFIS_ and FTJ_MFIM_, in contrast to the amorphous state retained in ReMem. Both PUND (100 kHz triangular pulses) and DC *I–V* measurements were conducted at RT, where the DC sweeps were carried out with Δ*V* = 50 mV and *I*_comp_ = 1 mA.

**Supplementary Figure S4.** (a, b) *I-V* curves of FTJ_MFIS_ and FTJ_MFIM_. FTJ_MFIS_ shows three distinct current steps—TAT leakage, polarization switching, and resistive switching— and (a) includes an additional unidirectional double sweep (0 to 5 V) to suppress polarization reversal, whereas FTJ_MFIM_ exhibits only polarization switching, reflecting the absence of oxygen vacancy migration. (c–e) Schematic band diagrams illustrating (c) TAT, (d) polarization switching, and (e) resistive switching.


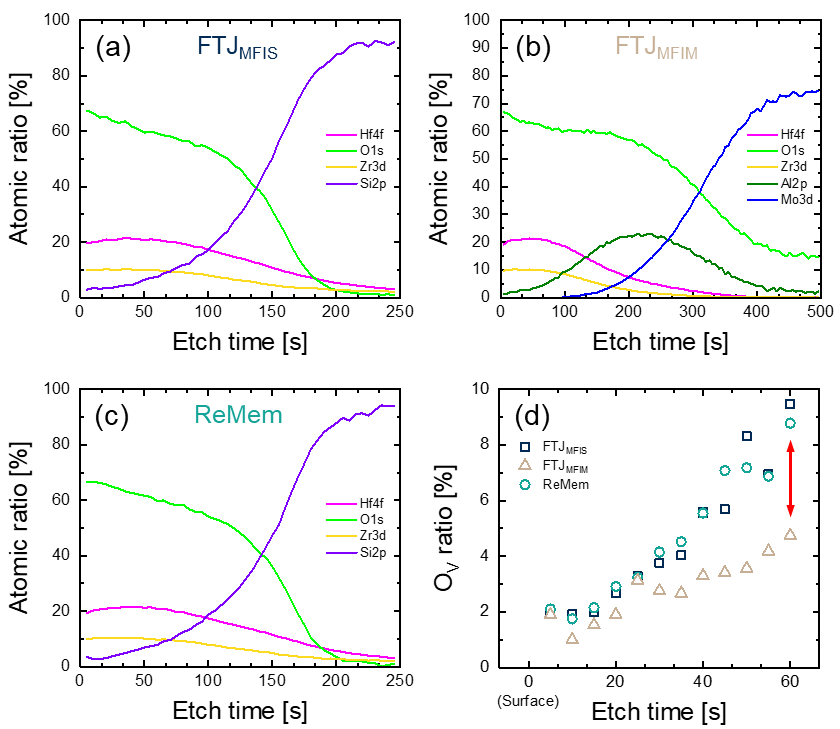


**Supplementary Figure S5.** (a–c) XPS depth profiles of FTJ_MFIS_, FTJ_MFIM_, and ReMem showing elemental distributions of Hf, Zr, O, Si, Mo, and Al. (d) Extracted oxygen vacancy ratio shows that FTJ_MFIS_ and ReMem maintain V_O_ content over 8 % near the surface, while FTJ_MFIM_ remains below ~4 %, consistent with switching characteristics.

**Supplementary Figure S6.** (a, c) Temperature-dependent *I-V* curves of FTJ_MFIS_ and FTJ_MFIM_. FTJ_MFIS_ shows increased TER despite decreasing *P*_r_, while FTJ_MFIM_ remains nearly unchanged. (b, d, e, g) *P-V* and *I-V* curves shows a temperature-induced decrease in *P*_r_ for FTJ_MFIS_ and an increase for FTJ_MFIM_, supporting the opposing MW trends. (f, h) F-N tunneling plots (ln(*I*/*E*^2^) versus 1/*E*) used to extract the effective barrier height: for FTJ_MFIS_​, linear fitting in the high-field region over *V*_READ_ = 0–4.3 V (ΔV = 50 mV) yields *Φ*_B_​=0.43–0.48 eV; for FTJ_MFIM_​, fitting over *V*_READ_ ​= 0–3.9 V (ΔV = 50 mV) yields *Φ*_B_ = 0.73–0.78 eV. (i, k) P–F plot (ln(*I*/*E*) versus *E*^1/2^) for FTJ_MFIS_ and FTJ_MFIM_. (j, l) P–F emission analyses, where the temperature-dependent linearizations (ln(*I*/*V×T^3^*^/2^) versus 1000/*T*) are used to extract the trap energy level: for FTJ_MFIS_​, *Φ*_T_ = 0.524–0.596 eV extracted within the P–F read window of *V*_READ_ ​= 4.0–4.3 V (Δ*V* = 100 mV); for FTJ_MFIM_​, *Φ*_T_ ​= 0.397–0.471 eV extracted within *V*_READ_ = 3.6–3.9 V (Δ*V* = 100 mV). FTJ_MFIS_ exhibits temperature-dependent variation, suggesting V_O_-assisted conduction, whereas FTJ_MFIM_ maintains a constant slope, confirming F-N tunneling as the dominant mechanism. Both PUND (100 kHz triangular pulses) and DC *I–V* measurements were conducted at 25, 45, 65, 85°C, where the DC sweeps were carried out with Δ*V* = 50 mV and *I*_comp_ = 1 mA.

**Supplementary Figure S7.** Maximum TER ratio as a function of *V*_Max_ for FTJ_MFIS_ and FTJ_MFIM_. FTJ_MFIS_ shows an abrupt increase in TER above 4.5 V due to resistive switching, while FTJ_MFIM_ shows gradual saturation. All Max(TER ratio)s were extracted at *V*_READ_ = 2 V.

**Supplementary Figure S8.** (a) *P-V* curves of FTJ_MFIIM_s with various bottom electrodes (TiN, W, Mo, Ti). FTJ_MFIIM_(Ti) show enhanced ferroelectricity. (b) *I-V* curves of FTJ_MFIIM_(Mo) under varying *V*_TOP_, showing polarization saturation followed by breakdown beyond 4.4 V. (c) *I–V* curves of FTJ_MFIIM_s with different bottom electrodes (Mo, W, TiN) measured at RT with *I*_comp_ = 1 mA and Δ*V* = 50 mV, exhibiting nearly identical leakage characteristics and breakdown voltages around 4.3–4.4 V.

**Supplementary Figure S9.** *I–V* curves of FTJ_MFIIM_(Ti) show no polarity-dependent change in TER on 5 samples (Δ*V* = 50 mV and *I*_comp_ = 1 mA).

**Supplementary Figure S10.** (a) *I–V* curves of FTJ_MFIIM_(Ti) measured at RT with increasing maximum sweep voltage (*V*_Max_), including the threshold voltages (*V*_th,1_ and *V*_th,2_) and the current gain (log_10_(*I*_LRS_/*I*_HRS_) at *V*_READ_ = 2 V). (b) Compact *V*_th_ map summarizing the distributions of *V*_th,1_ and *V*_th,2_ with operating regions marked as polarization switching, hybrid switching, and risk. (c) Current gain–*V*_Max_ characteristics extracted from 16 samples using incremental step-stress.


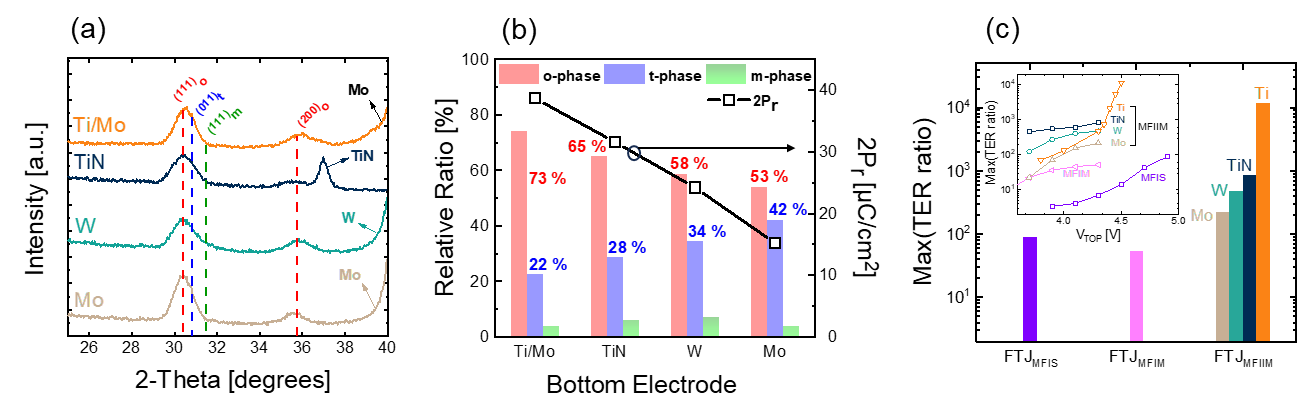


**Supplementary Figure S11.** (a) XRD spectra of FTJ_MFIIM_s with different bottom electrodes. (b) Proportion of o-, t-, and m-phases and corresponding 2*P*_r_ values in FTJ_MFIIM_s. A clear correlation is observed between o-phase ratio and 2*P*_r_. (c) Maximum TER ratios extracted at optimal *V*_TOP_, with the FTJ_MFIIM_(Ti) showing the highest value of ~10^4^.


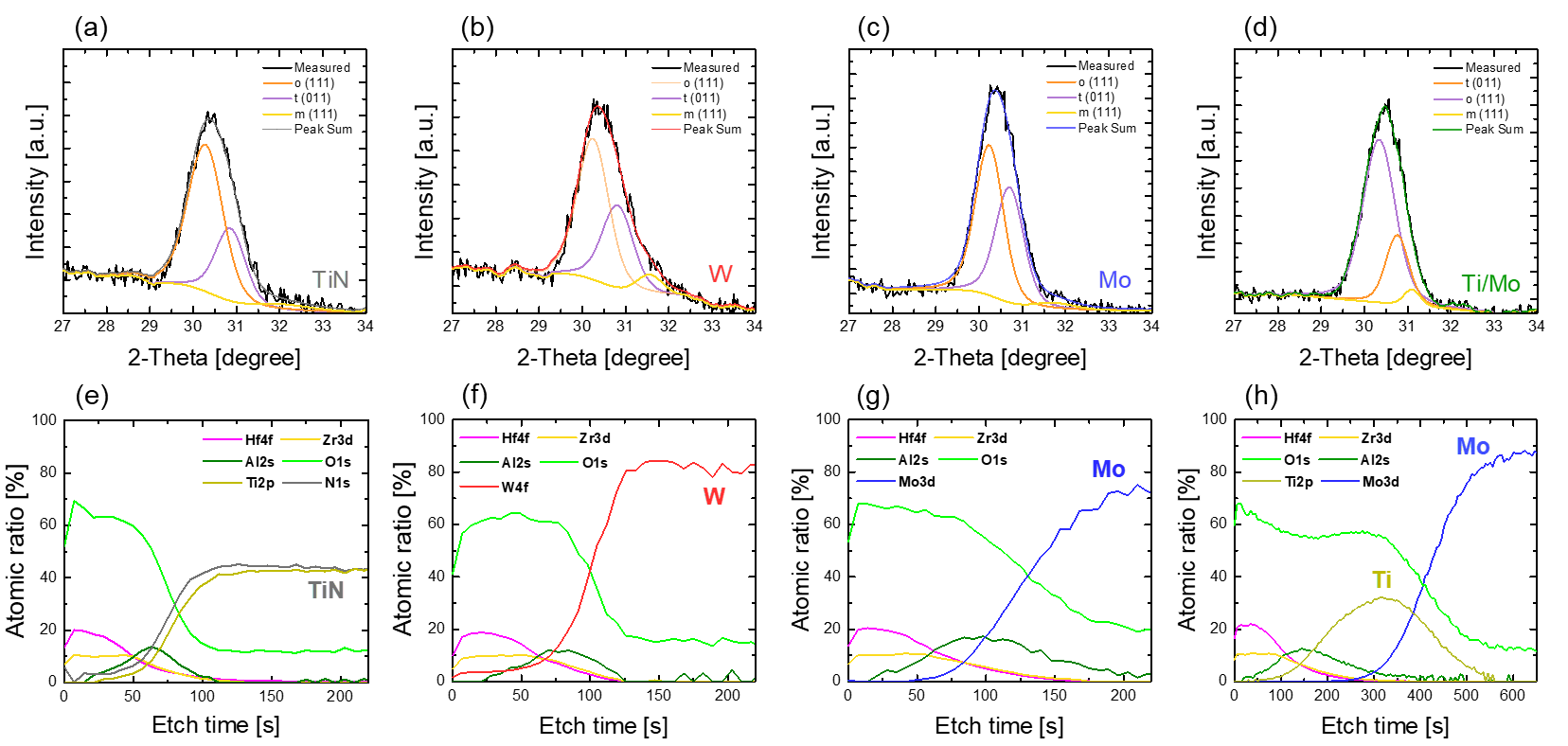


**Supplementary Figure S12.** (a–d) XRD peak deconvolution results of HZO films on different bottom electrodes (TiN, W, Mo, Ti/Mo). (e–h) XPS depth profiles of FTJ_MFIIM_s, revealing Hf, Zr, O, Al, Mo, Ti, N, and W.

**Supplementary Figure S13.** Temperature-dependent linearizations for a representative FTJ_MFIIM_(Ti). (a) F–N plot (ln(*I/E^2^*) versus 1/*E*) measured over *V*_READ_​=0–4.5 V. (Δ*V*_READ_ ​= 50 mV) The effective barrier height extracted from the F–N slopes is *Φ*_B_ ​= 1.21–1.26 eV. (b, c) Temperature-dependent P–F plot used to extract the trap energy level *Φ*_T_​, plotted as ln(*I/V·T*^3/2^) versus 1000/*T* for polarization and hybrid switching region: *V*_READ_​=3.5–3.8 V (Δ*V* = 100 mV) in (b) and *V*_READ_ ​= 4.3–4.5 V (Δ*V* ​= 50 mV) in (c), yielding *Φ*_T_ = 0.336–0.417 eV and 0.324–0.381 eV, respectively. (d,e) P–F plots (ln(*I/E*) versus *E*^1/2^) in the polarization switching region (*V*_READ_ = 3.5–3.8 V, Δ*V* = 100 mV) in (d) and the hybrid switching region (*V*_READ_ ​= 4.3–4.5 V, Δ*V* ​= 50 mV) in (e), measured at 25, 45, 65, and 85 °C.

**Table S1.** Material parameters (thickness, Φ, χ, and κ) used for the FTJs.

**Supplementary Figure S14.** Effective ferroelectric field (*E*_FE_) conversion and regime interpretation for the FTJ_MFIS_​, FTJ_MFIM_​, FTJ_MFIIM_​, and FTJ_MFIIM_(Ti)​. The applied *V*_total_ is converted to *E*_FE_ using a series-capacitance model based on the material parameters summarized in Table S1.


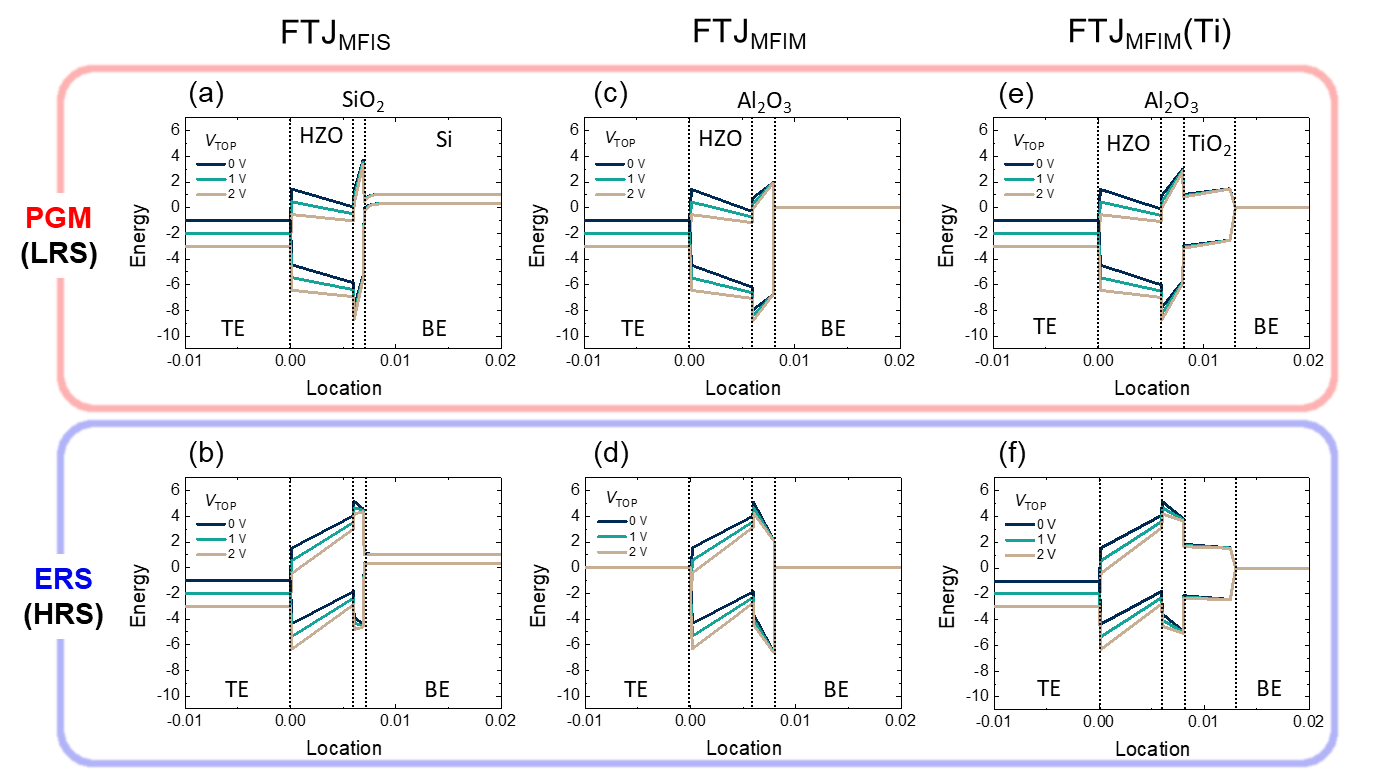


**Supplementary Figure S15.** Simulated energy band diagrams of FTJ_MFIS_, FTJ_MFIM_, and FTJ_MFIIM_(Ti) under program (a, c, e) and erase (b, d, f) states, showing barrier modulation with increasing *V*_TOP_. Simulations were performed using Sentaurus TCAD with Preisach polarization model.

**Supplementary Figure S16.** (a-d) Switching characteristics of FTJ_MFIIM_(Ti) under varying *V*_PGM_ and *V*_ERS_ (*V*_READ_ = 2 V and *I*_comp_ = 1 mA), showing distinct switching mechanism from polarization to resistive switching. (e, f) Endurance test up to 10^8^ cycles confirms stable operation with minimal degradation. (g, h) Retention performance at 25 °C showing stable LRS and HRS over 10^4^ s; FTJ_MFIIM_(Ti) exhibits slight current decay within few seconds due to V_O_ redistribution. (i) Pulse scheme diagram including write-read sequence used for switching speed, endurance, and retention measurements.


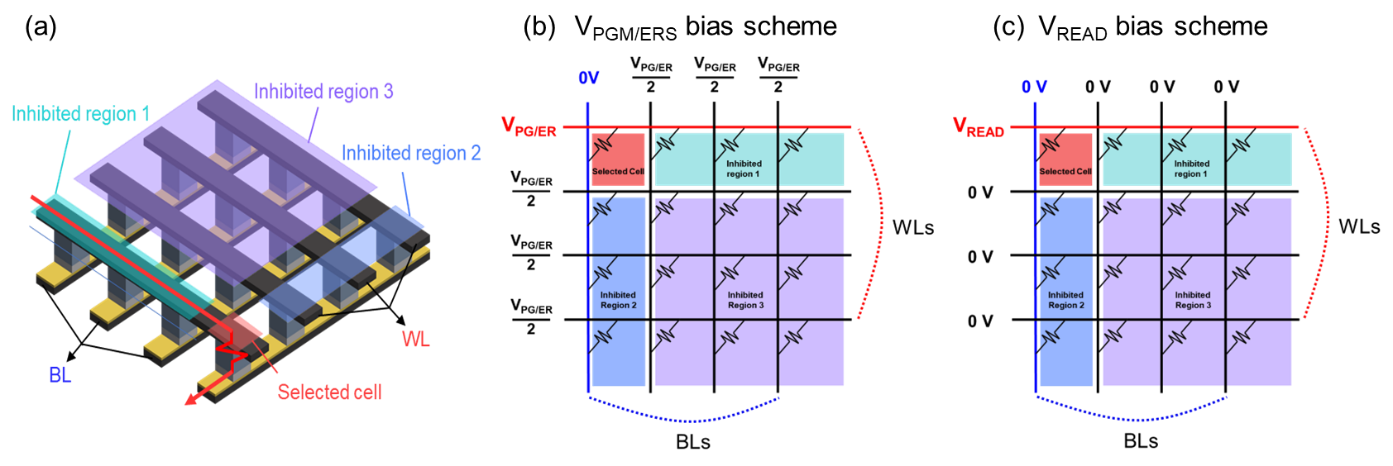


**Supplementary Figure S17.** (a) Schematic of FTJ array shows selected/inhibited regions during write/read operations. (b, c) Applied bias schemes for *V*_PGM/ERS_ and *V*_READ_ operations, enabling precise cell selection while suppressing unintended switching in unselected lines through *V*_PGM/ERS_/2 biasing.

**Supplementary Figure S18.** (**a)** *I–V* characteristics of the FTJ array at RT in different resistance states. **(b)** D2D variation of 25 FTJ cells. Distinct current levels between two resistance states are clearly observed at *V*_READ_ = 2 V, while negligible current variation occurs at 0 V, confirming stable operation and reproducible device characteristics.


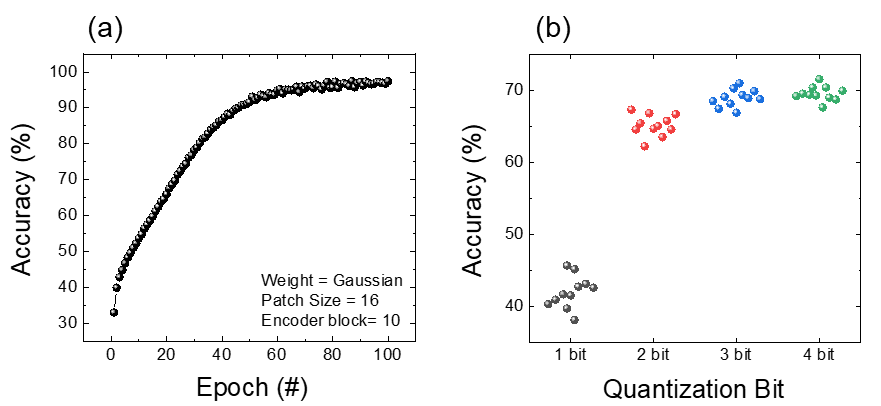


**Supplementary Figure S19.** (a) Prediction accuracy of a ViT model trained over 100 epochs on the CIFAR-10 dataset. (b) Inference accuracy under different quantization bit widths, evaluated over 10 independent runs. As the bit width decreases, particularly at 1-bit quantization, the accuracy shows increased variance due to significant information loss.


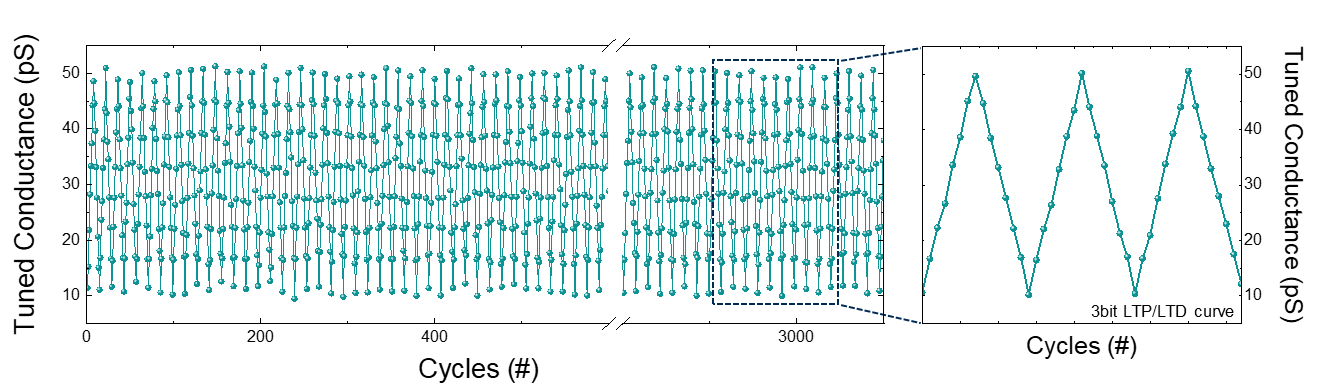


**Supplementary Figure S20.** Multi-level tuning characteristics of 3-bit conductance states, evaluated through iterative programming cycles.


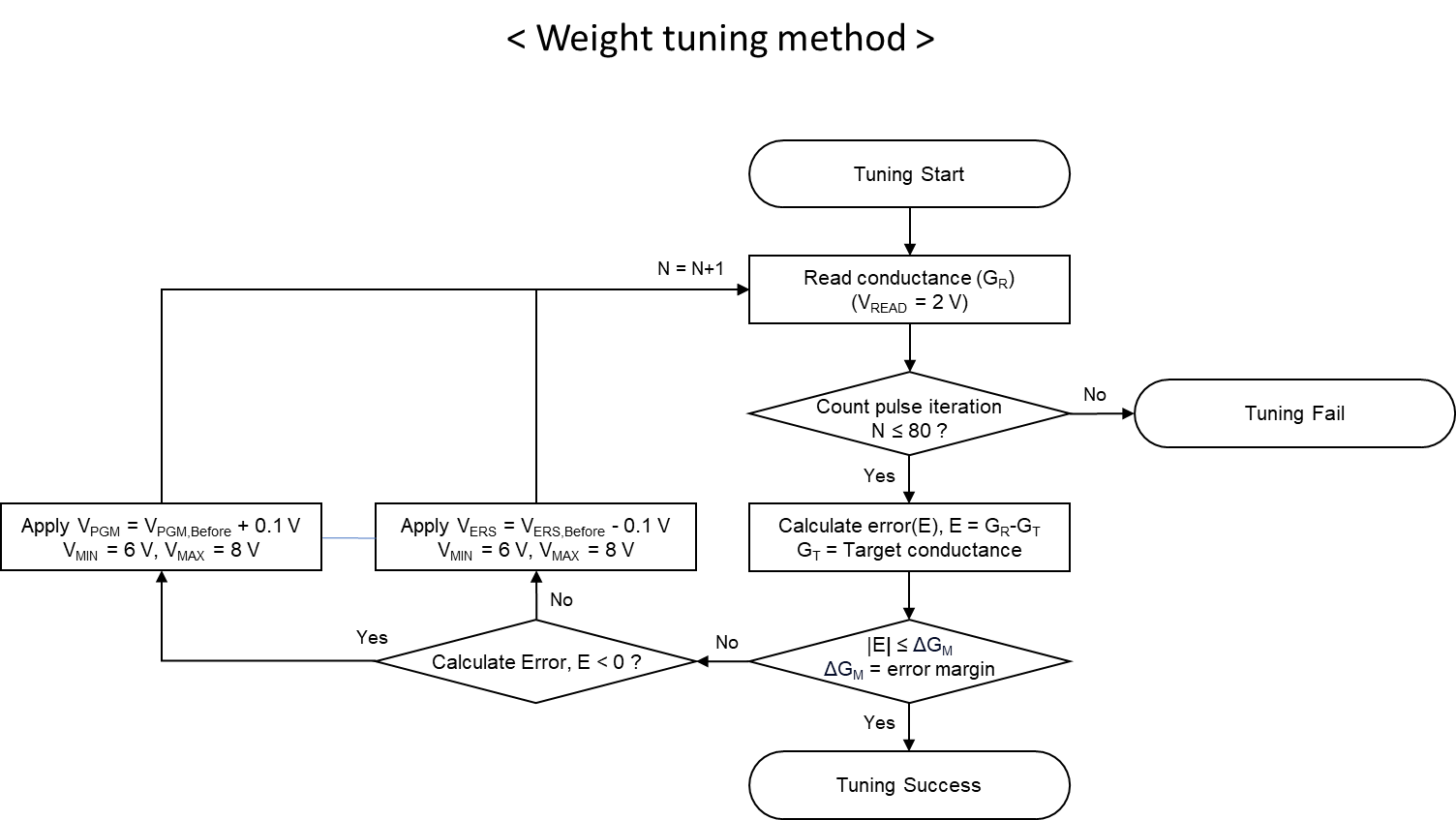


**Supplementary Figure S21.** Weight tuning scheme for the FTJ array using a LabVIEW-based system with integrated Keysight instruments. Selected cells are programmed with ±6 V, 1 μs pulses and fine-tuned via ISPP in 0.1 V steps. Tuning ends when the read conductance (*G*_R_) satisfies |*G*_R_ - *G*_T_| < Δ*G*_M_. *V*_READ_ = 2 V is used for non-destructive reads, and non-selected cells are inhibited to suppress disturbance.

**Supplementary Figure S22.** Target and transferred synaptic weights for (a) query, (b) key, and (c) value components under *G*⁺ and *G*⁻ conditions. A fine-tuning algorithm with a 20 % tuning margin enables accurate weight transfer across the FTJ arrays.

**
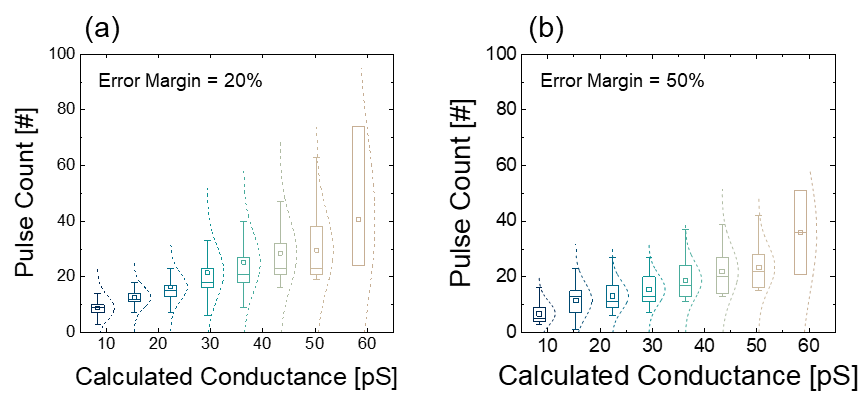
**

**Supplementary Figure S23.** Number of programming pulses required to reach each conductance level under different tuning margin conditions ((a) 20 %, (b) 50 %). As the tuning margin increases, the number of pulses required per level decreases.

**
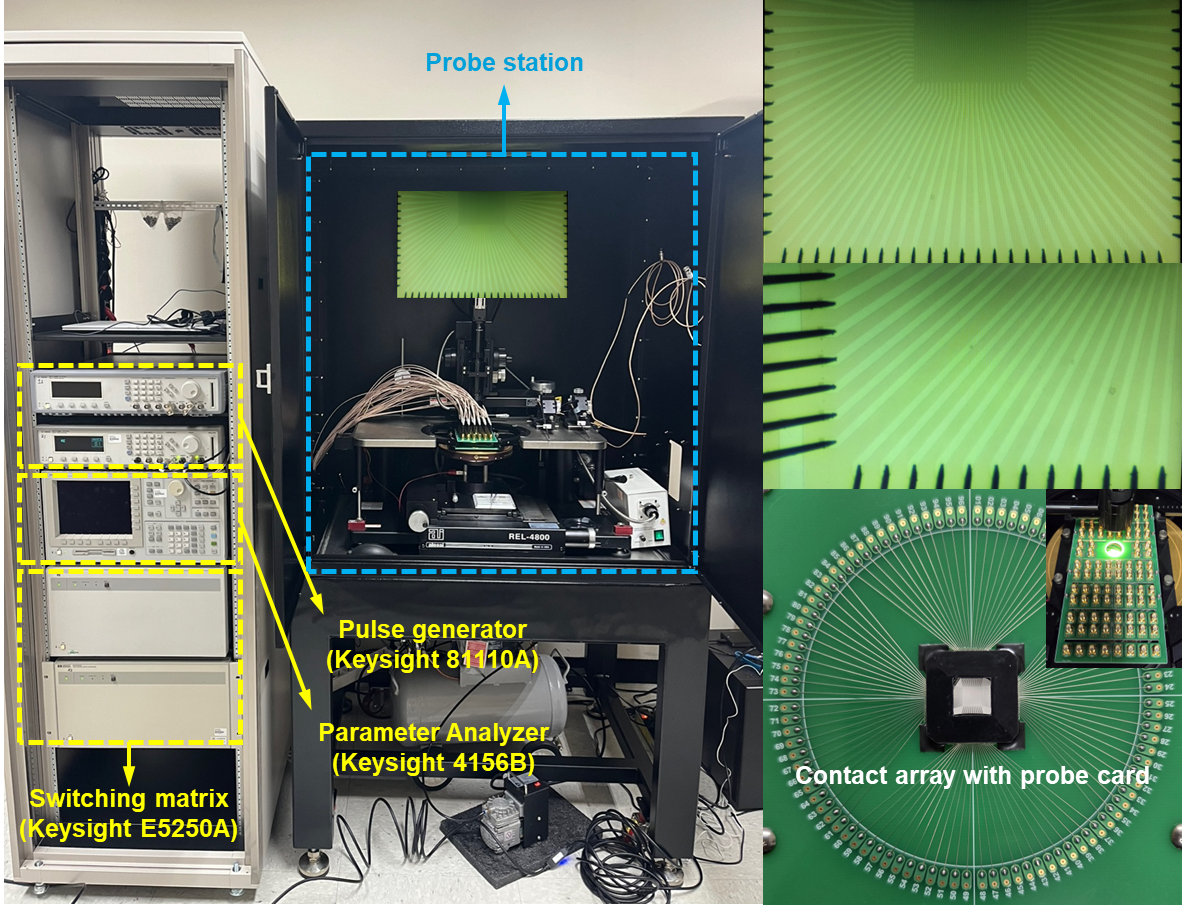
**

**Supplementary Figure S24.** Measurement setup for FTJ array characterization, including Keysight 81110A pulse generator, 4156B parameter analyzer, and E5250A switching matrix. The probe station is connected to the array through a probe card interfacing with contact pads.
